# Hemiurid Trematodes (Digenea: Hemiuridae) from Marine Fishes off the Coast of Rio de Janeiro, Brazil, with Novel Molecular Data

**DOI:** 10.3390/ani12233355

**Published:** 2022-11-29

**Authors:** Camila Pantoja, Olena Kudlai

**Affiliations:** Institute of Ecology, Nature Research Centre, Akademijos 2, 08412 Vilnius, Lithuania

**Keywords:** Aphanurinae, Dinurinae, Hemiurinae, Lecithochirinae, marine fish, mitochondrial and nuclear DNA, southwestern Atlantic Ocean

## Abstract

**Simple Summary:**

Brazil, with its remarkably diverse marine habitats, harbour one of the world’s richest fish diversities. Consequently, the diversity of their trematode parasites is also expected to be extremely rich. However, our current knowledge on this group of animals is incomplete and there are many unknown trematode species that await discovery and genetic characterisation. The Hemiuridae (Digenea) is the second most speciose trematode family in marine fishes from Brazil; however, to date, it remains understudied. We examined forty-three specimens of nine fish species belonging to eight families (Carangidae, Clupeidae, Haemulidae, Muraenidae, Percophidae, Pinguipedidae, Trichiuridae, and Triglidae) collected from the coastal zone off Rio de Janeiro, Brazil and found hemiurid trematodes in the stomach of 14 fishes. Using morphological and molecular analyses, we identified eight species from four genera of the family Hemiuridae. One of these species is reported in Brazil for the first time, four are reported from new fish hosts, and four were genetically characterised for the first time. Our novel data contributes to the knowledge on marine biodiversity in Brazil and will further contribute to the classification of the family Hemiuridae.

**Abstract:**

Brazil is a tropical country with remarkably diverse marine habitats that harbour a rich diversity of fish. Only a small portion of this fish diversity has been investigated for parasites, and thus the diversity of their trematode parasites remains unexplored. Moreover, only 5 out of 184 known digenean trematode species of marine fish in Brazil have been genetically characterised. The Hemiuridae Looss, 1899 is the second most speciose trematode family in marine fishes from Brazil but, in many ways, it remains a neglected group. Forty-three trematode specimens from nine fish species were collected from the coastal zone off Rio de Janeiro, Brazil. Trematodes were found in the stomach of 14 specimens of 9 fish species belonging to 8 families (Carangidae, Clupeidae, Haemulidae, Muraenidae, Percophidae, Pinguipedidae, Trichiuridae, and Triglidae). Trematode specimens were studied using morphological and molecular genetic analyses. A total of eight hemiurid species from four genera, *Ectenurus*, *Lecithochirium*, *Myosaccium*, and *Parahemiurus* were identified. This paper reports on new host records for four species of hemiurids, adds a new record on the geographical distribution for one species, and provides the first DNA sequence data supplemented with the detailed description of morphology for five species. Phylogenetic analyses supported that the subfamily classifications of the Hemiuridae—based entirely on morphological characters—needs to be reconsidered, taking into account a wider range of information sources.

## 1. Introduction

Brazil is a tropical country with remarkably diverse marine habitats that harbour one of the world’s highest diversities of fish [1,2]. Based on the assumption that parasite diversity is positively correlated with host diversity [3], parasites ought to be one of the specious components of Brazilian marine fauna. Since the first report on helminth parasites in marine fishes in Brazil in 1819, the discovery of these parasites continues to accelerate, with digenean trematodes shown to be one of the most diverse parasite groups [4,5]. However, our knowledge on the actual species diversity of digenean trematodes, their host associations, life cycles, distributions, and phylogenetic relationships remains limited [6,7]. Furthermore, and despite continued interest in this group of parasites, the diversity of digenean trematodes is not reflected in the available genomic data from Brazil. To date, there are only five species for which DNA sequence data is available [7].

The Hemiuroidea is possibly the most ubiquitously distributed superfamily of cosmopolitan and morphologically diverse digeneans that predominantly parasitise marine teleost fish [8]. In Brazil, members of 11 hemiuroid families were reported from phylogenetically and ecologically diverse marine teleosts, belonging to 32 families of 20 orders [4,9]. Out of 16 currently recognised families within the Hemiuroidea, the Hemiuridae Looss, 1899 is one of the most speciose but, in many ways, remains a neglected group. Despite numerous described species of hemiurids, the morphological descriptions of many are inadequate and numerous species therefore require re-evaluation and redescription. Although hemiuroids were one of the first superfamilies to be phylogenetically analysed based on DNA sequences data, the limited number of taxa used in historic and recent studies [10,11,12] do not allow for the clarification of the statuses of several families and subfamilies. Additionally, the identification of some isolates used for sequence generation remain questionable and there are no vouchers available to re-evaluate the material. Similarly, the lack of DNA sequences for numerous taxa within the Hemiuridae hinders the elucidation of systematics of the family based on molecular phylogenetic analysis. Therefore, the taxonomic identity of many taxa within the family are still based entirely on morphological data [9].

Hemiurid life cycles are complex and often involve the exploitation of three to four hosts. However, to date, complete or fragmentary data is only available for 13 species [13]. In Brazil, the life cycle was partially elucidated for a species from the genus *Parahemiurus*, *Parahemiurus merus* [14].

The Hemiuridae is the second most species-rich family among fish digeneans in marine ecosystems in Brazil [9]. A total of 28 species from 10 genera are known to parasitise fishes from 28 families of 17 orders in Brazil [7,9]. The majority of species were reported from fishes of the family Carangidae (Carangiformes) collected off the Rio de Janeiro coast [9,15,16]. Only recently were the first DNA sequences of Brazilian marine digenean trematodes published, with three of them being of hemiurids [7].

Parasitological examination of fish for the present study was carried out in the State of Rio de Janeiro, Brazil in January 2021. Overall, the most frequent digeneans collected were species of the family Hemiuridae. They were found in nine species of marine fishes. Here, we provide detailed descriptions of the morphology of the recorded hemiurids, together with newly generated DNA sequences. Furthermore, in order to assess the subfamilial/generic affiliations and relationships of the studied species, we carried out phylogenetic analyses using newly generated sequences of the 28S ribosomal RNA gene. Based on novel data generated in this study, we assessed the phylogenetic affinities for three hemiuroid genera, namely, *Ectenurus*, *Myosaccium*, and *Parahemiurus*, for the first time.

## 2. Materials and Methods

### 2.1. Host and Parasite Collection and Morphological Evaluation

In January 2021, forty-three specimens of nine species of fish, namely, *Anisotremus virginicus* (Linnaeus) (Haemulidae), *Decapterus punctatus* (Cuvier) (Carangidae), *Gymnothorax vicinus* (Castelnau) (Muraenidae), *Harengula clupeola* (Cuvier) (Clupeidae), *Percophis brasiliensis* Quoy and Gaimard, 1825 (Percophidae), *Prionotus punctatus* (Bloch) (Triglidae), *Pseudopercis numida* Miranda Ribeiro (Pinguipedidae), *Sardinella brasiliensis* (Steindachner) (Clupeidae), and *Trichiurus lepturus* (Linnaeus) (Trichiuridae) were received from local fisherman from the coastal zone of Cabo Frio (22°52′46″ S, 42°01′07″ W), State of Rio de Janeiro, Brazil. Fish taxonomy and names were used in accordance with Froese and Pauly [2]. Fish were examined for the presence of infection with helminth parasites. Trematode individuals collected from examined fish were washed in 0.9% saline, heat killed, and fixed in 96% ethanol and in 4% formalin (further hologenophores and paragenophores sensu Pleijel et al. [17]). Specimens selected for molecular analyses (i.e., hologenophores) were processed as described in Faltýnková et al. [18], i.e., a small piece of the body of each specimen was excised and used for DNA extraction and the remaining voucher (hologenophore) was used for morphological evaluation. Specimens fixed in formalin (i.e., paragenophores) and the remaining vouchers were stained in Mayer’s hydrochloric carmine solution and mounted as described in Pantoja et al. [7], and thereafter, used for morphological evaluation. Drawings were made by use of a drawing attachment to a light microscope Olympus BX 51. Measurements were taken using AxioCam image analysis software adapted to a Zeiss Primo Star light microscope and, unless otherwise stated, are given in micrometres (μm). Voucher specimens are lodged in the Helminthological Collection of the Oswaldo Cruz Institute (CHIOC), Rio de Janeiro, Brazil.

### 2.2. DNA Sequence Generation and Phylogenetic Analyses

Total genomic DNA was extracted from trematodes and fish (from a fin clipping) following Georgieva et al. [19]. For trematodes, DNA amplification of two partial fragments of the mitochondrial *cox*1 gene was performed using the primers JB3 (forward; 5′-TTT TTT GGG CAT CCT GAG GTT TAT-3′) (Bowles et al. [20]) and CO1-R-trema (reverse; 5′-CAA CAA ATC ATG ATG CAA AAG G-3′) (Koehler et al. [21]), and Dig_cox1Fa (forward; 5′-ATG ATW TTY TTY TTY YTD ATG CC-3′) and Dig_cox1R (reverse; 5′-TCN GGR TGH CCR AAR AAY CAA AA-3′) (Wee et al. [22]) following the polymerase chain reaction (PCR) protocols as described in Koehler et al. [21] and Wee et al. [22], respectively. The D1–D3 region of the large ribosomal subunit 28S rDNA gene was amplified using the primers digl2 (forward; 5′-AAG CAT ATC ACT AAG CGG-3′) and 1500R (reverse; 5′-GCTA TCC TGA GGG AAA CTT CG-3′) (Snyder and Tkach, [23]), as well as of the ribosomal ITS1-5.8S-ITS2 region were generated using the primers D1 (forward; 5′-AGG AAT TCC TGGTAA GTG CAA G-3′) and D2 (reverse; 5′-CGT TAC TGA GGG AAT CCT GGT-3′) (Galazzo et al. [24]) following the PCR protocols as described in Tkach et al. [25] and Galazzo et al. [24], respectively. The ribosomal ITS2 region was amplified using the primers 3S (forward; 5′-GGT ACC GGT GGA TCA CGT GGC TAG TG-3′) (Bowles et al. [26]) and ITS2.2 (reverse; 5′-CCT GGT TAG TTT CTT TTC CTC CGC-3′) (Cribb et al. [27]) following the PCR protocol as described in Cutmore et al. [28]. For fish, DNA amplification of the partial mitochondrial *cox*1 gene was performed using the primers VF1 (forward; 5′-TCT CAA CCA ACC ACA AAG ACA TTGG-3′) and VR1 (reverse; 5′-TAG ACT TCT GGG TGG CCA AAG AAT CA-3′) [29] following the PCR protocol of Ward et al. [29]. PCR amplicons were purified with the Exo-SAP-IT KitTM Express Reagent (Thermo Fisher Scientific Baltics UAB, Vilnius, Lithuania) and sequenced using the Big Dye Terminator V3.1 Cycle Sequencing kit and ABI 3730 (XL) DNA analyzer capillary sequencing robot (Applied Biosystems, Foster City, CA, USA). The original PCR primers were used for sequencing. Two additional internal primers 300F (forward; 5′-CAA GTA CCG TGA GGG AAA GTT G-3′) (Littlewood et al. [30]) and ECD2 (reverse; 5′-CCT TGG TCC GTG TTT CAA GAC GGG-3′) (Littlewood et al. [31]) were used for sequencing of the 28S rDNA amplicons. Geneious ver. 11 (Biomatters, Auckland, New Zealand) was used to assemble and edit sequences. The newly generated sequences (*cox*1, 445–500 nt and 756 nt for trematodes; *cox*1 620–677 for fish; ITS1-5.8S-ITS2, 1676 nt; ITS2, 779–782 nt; and 28S rDNA, 1181–1268 nt) were deposited in GenBank with accession numbers OP918119–OP918132; OP918136–OP918139; OP918021–OP918026; OP948302–OP948305 for trematodes and OP905634; OP925860 for fish.

Three alignments, including novel and previously published sequences, for the species of the Hemiuridae were built using ClustalW implemented in Geneious ver. 11. All alignments were used for comparative sequence analysis (p-distance and nucleotide [nt] difference) and Alignment 1 was further used for phylogenetic analyses. Alignment 1 (957 nt long) included 37 28S rDNA sequences of the species from the Hemiuridae; 14 sequences of 7 species generated in the present study. Alignment 2 (416 nt long) included six ITS2 sequences of *Lecithochirium* spp.; three sequences of three species generated in the present study. Alignment 3 (443 nt long) included eight *cox*1 sequences of four species of *Lecithochirium*; six sequences of four species generated in the present study. Pairwise genetic distances (uncorrected p-distance and no. of differences) for the three datasets were calculated in MEGA ver. 11 [32] using the following conditions: “Variance Estimation Method = None”, “Model/Method = p-distance or no. of differences”, “Substitutions to Include = d: Transitions + Transversions” and “Gaps/Missing Data Treatment = Pairwise deletion”.

Phylogenetic relationships of the taxa in Alignment 1 were assessed using Bayesian inference (BI) and maximum likelihood (ML) analyses. Sequences of *Isoparochis eurytremus* (MH628315), a parasite of *Silurus asotus* Linnaeus from Takashima, Japan, was used as the outgroup based on the topology in the phylogenetic tree of the superfamily Hemiuroidea by Louvard et al. [13]. The analyses were conducted using the GTR + I + G model, which was predicted as the best model by the Akaike Information Criterion in jModelTest 2.1.2 [33]. BI analysis was performed using MrBayes software (ver. 3.2.3) [34] through the CIPRES Science Gateway ver. 3.3 [35] accessed on 22 September 2022. Markov Chain Monte Carlo chains were run for 10,000,000 generations, log-likelihood scores were plotted and only the final 75% of trees were used to build the consensus tree. ML analysis was performed using PhyML ver. 3.0 [36] and run on the ATGC bioinformatics platform (http://www.atgc-montpellier.fr, accessed on 22 September 2022) with a nonparametric bootstrap value of 100 pseudoreplicates. 

To avoid ambiguity for some generic names, the following abbreviations were used: D., *Decapterus*; Di., *Dinurus*; H., *Harengula;* He., *Hemiurus;* O., *Oligoplites;* Op., *Opisthonema;* P., *Parahemiurus;* Pe., *Percophis*; Pr., *Prionotus*; Ps., *Pseudopercis*; Pu., *Pulmovermis*; S., *Sardinella*; and Sa., *Sardinops*.

## 3. Results

Evaluation of the specimens of hemiurid trematodes obtained in the present study via morphological and molecular methods confirmed the presence of eight trematode species in the examined fishes. Out of the 43 specimens of 9 fish species examined, 14 (32.5%) were found to be infected with at least 1 species of hemiurid and 4 (9.3%) were infected with 2 species of hemiurids. All specimens of hemiurids were detected in the fish stomach. Thirty sequences were newly generated; twenty-eight sequences for seven out of eight recorded species of trematodes: 28S rDNA (*n* = 14), ITS1-5.8S-ITS2 (*n* = 1), ITS2 (*n* = 3) and *cox*1 (*n* = 10). Sequences of the unidentified species of *Lecithochirium* were not generated. Two *cox*1 sequences were generated for the fish hosts, *Pseudopercis numida* and *Trichiurus lepturus*. The photographs of the fish specimens that were used to generate sequences are provided in Figure S1. Fish hosts were identified following Menezes and Figueiredo [37]. The *cox*1 sequences for fish obtained in the present study were compared with sequences available in GenBank. The sequence divergence between our specimen of *T. lepturus* (OP905634) and specimens of *T. lepturus* (GU702467; JX124915) collected in São Paulo, Brazil was low and ranged from 0.2 to 0.3% (1–2 nt). Thus, we consider these isolates as conspecific. The sequence divergence between our specimen of *Ps. numida* (OP925860) and specimens of *Ps. semifasciata* (JQ365526; EU074573) from São Paulo, Brazil and from Argentina was low and ranged from 0 to 1.6% (0–8 nt). Although the genetic divergence between our specimen identified as *Ps. numida* and specimens of *Ps. semifasciata* available in GenBank suggests that these specimens are conspecific, morphologically our specimens belong to *Ps. numida*.

### 3.1. Morphological Evaluation

**Aphanurinae** **Skrjabin and Guschanskaja, 1954**
***Myosaccium*** **Montgomery, 1957**
***Myosaccium*** ***ecaude*** **Montgomery, 1957**
*Host*: *Sardinella brasiliensis* (Steindachner) (Clupeidae).
*Infection rates*: two out of two; three–four specimens per fish; seven specimens in total.
*Representatives DNA sequences*: OP918123 (28S); OP948303 (*cox*1).
*Voucher material*: CHIOC 39908 a–e.

**Figure 1:**
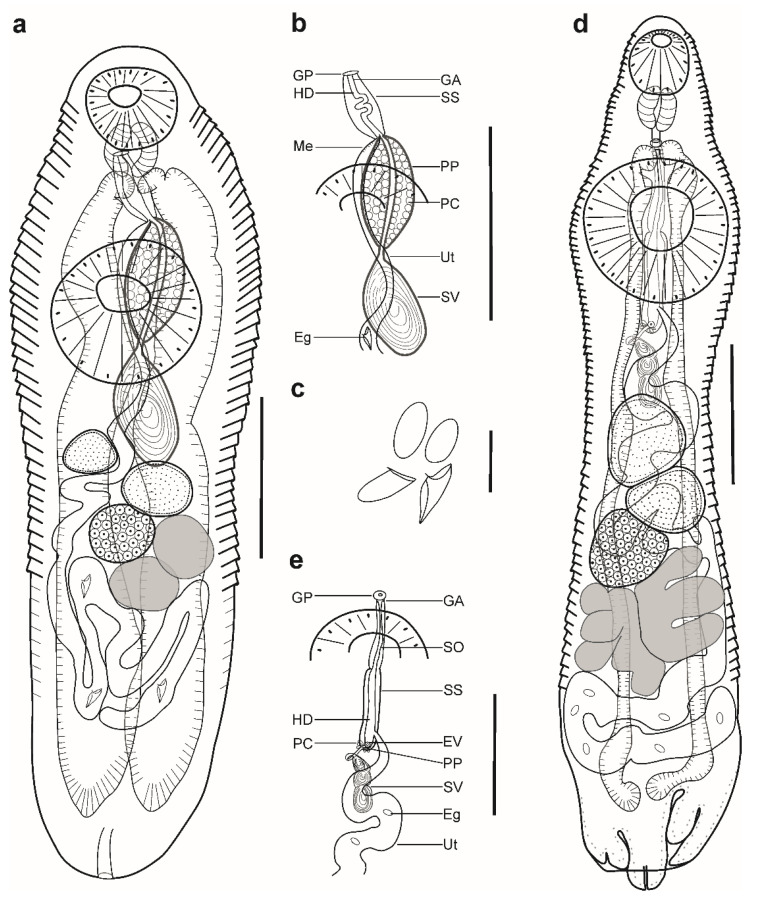
Hemiurid parasites of marine fishes from Brazil. (**a**–**c**) Adult of *Myosaccium ecaude* ex *Harengula clupeola*: (**a**) complete specimen, ventral view; (**b**) detail of the terminal genitalia, ventral view; (**c**) eggs; (**d**,**e**) adult of *Ectenurus virgula* ex *Decapterus puntatus*: (**d**) complete specimen, ventral view; (**e**) detail of the terminal genitalia, ventral view. *Scale bars* (**a**,**b**,**d**,**e**): 200 µm; (**c**): 50 µm. *Abbreviations*: Eg, eggs; GA, genital atrium; GP, genital pore; HD, hermaphroditic duct; PC, prostatic cells; PP, pars prostatica; SO, sinus-organ; SS, sinus-sac; SV, seminal vesicle; Ut, uterus.

Description (Figure 1a–c). (Based on four paragenophores and one hologenophore; measurements of paragenophores in Table 1 and hologenophore in description): Body elongate, dorso-ventrally flattened. Maximum width at level of ventral sucker, 189. Tegument covered with conspicuous plications to level of vitellarium. Forebody short 217 long.

Pre-oral lobe distinct, 25 long. Oral sucker muscular, well developed, subspherical or transversely oval, ventro-subterminal, 76 long, 86 wide. Prepharynx absent. Pharynx muscular, well developed, spherical or subspherical, 45 long, 45 wide. Oesophagus absent. “Drüsenmagen” present. Intestinal bifurcation just posterior to pharynx. Caeca blind, with thin walls and wide lumen, terminates in posterior body extremity. Ventral sucker muscular, well developed, spherical or subspherical, 129 long, 139 wide, larger than oral sucker (1:1.62), pre-equatorial.

Testes 2, obliquely symmetrical, separated, entire, pre-ovarian, in anterior hindbody, separated from ventral sucker; dextral testis subspherical to transversely oval, 32 long, 28 wide, sinistral testis transversely oval, 38 long, 55 wide. Distance between ventral sucker and dextral testis 50. Seminal vesicle thick-walled, saccular, elongate-oval, 128 long, 109 wide (Figure 1a,b). Seminal vesicle entirely in anterior hindbody or extends only to posterior margin of ventral sucker, connected to pars prostatica by aglandular duct. Pars prostatica elongate-oval, vesicular, densely invested by prostatic cells, antero-dorsal to ventral sucker (Figure 1b). Sinus-sac elongate, between suckers, with muscular wall, 100 long, 28 wide. Hermaphroditic duct straight or coiled within sinus-sac, opens directly through genital pore. Genital pore median or sub-median just posterior to oral sucker.

Ovary median, entire, subspherical (*n* = 3) or transversely oval (*n* = 2), 48 long, 68 wide, in anterior half or in middle of hindbody, contiguous with sinistral testis. Vitellarium in two compact oblique masses, subspherical; dextral mass posterior to ovary, 37 long, 41 wide, sinistral mass lateral to ovary, 41 long, 39 wide. Juel’s organ and Mehlis’ gland not observed. Uterus coiled, occupies post-ovarian region. Metraterm not differentiated, terminal part of uterus passes into sinus-sac ventrally, joins male duct forming hermaphroditic duct. Eggs numerous, 25–26 × 11–13 (*n* = 4) (Figure 1c).

Excretory vesicle not observed; excretory pore terminal.

***Remarks*:** Specimens found in the present study correspond well to the generic diagnosis of *Myosaccium* Montgomery, 1957 provided by Gibson [8] in having a saccular and elongate-oval seminal vesicle posterior to the middle of the ventral sucker, a vesicular pars prostatica with a muscular wall, a tubular sinus-sac enclosing the hermaphrodite duct, and vitellarium composed of two distinct masses.

Our specimens correspond in their morphology to the type-species of the genus, *M. ecaude* Montgomery, 1957, described from the stomach of South American pilchard, *Sardinops sagax* (=*S. caerulea*) (Jenyns) by Montgomery [38] in La Jolla, CA, USA, north-eastern Pacific Ocean. Specimens corresponded in body shape as well as by possessing plicated tegument, a subspherical pharynx, obliquely symmetrical testes, and a similar sucker ratio (1:1.66–1.79 vs. 1:1.57–1.75). However, the maxima for all dimensions in our specimens was lower except for the eggs (Table 1). Except for the sucker ratio (1:1.66–1.79 vs. 1:2.30) (Table 1), all dimensions of our specimens overlap with specimens of *M. ecaude* collected from *Opisthonema libertae* (Günther) in the Chamela Bay, Jalisco, Mexico, north-eastern Pacific Ocean by León-Règagnon et al. [39].

*Myosaccium ecaude* is known as a parasite of the stomach of fishes from the family Clupeidae in the Pacific and Atlantic oceans. After the original description, the species was reported in *Op. libertate* and *Harengula thrissima* (Jordan and Gilbert) in the Chamela Bay, Jalisco, Mexico [39,40], in the type host *Sa. sagax* in California, USA, and Baja California, Mexico, north-eastern Pacific Ocean [41,42,43] and in *H. clupeola* and *S. brasiliensis* in Rio de Janeiro, Brazil, southwestern Atlantic Ocean [44,45].

*Myosaccium ecaude* was originally described as possessing filamented eggs. Overstreet [46] and León-Règagnon et al. [39] re-evaluated the type material and provided different conclusions. The first author concluded that “specimens from *Sardinella anchovia* do not have filaments or spines on the eggs, although a look at collapsed specimens on a fixed plane strongly suggests their presence”. Additionally, the same author did not observe morphological differences between *M. ecaude* and the only other species of the genus, *M. opisthonemae* (Siddiqi and Cable, 1960) described from *Op. oglinum* (Lesueur) in Playa Mani, Puerto Rico, by Siddiqi and Cable [47], north-western Atlantic Ocean, and suggested that *M. opisthonemae* might be a small or progenetic form of *M. ecaude*. León-Règagnon with co-authors [39] observed the presence of filamented eggs in the type material and in their newly collected specimens from fish in Mexico. These authors suggested that the presence of filamented eggs is a strong characteristic to distinguish *M. ecaude* from *M. opisthonemae*. Thereafter, Kohn and Buhrnheim [48] collected specimens of *M. ecaude* in *S. aurita* in the same region to that of the present study and described their specimens as having filamented and spined eggs. However, later Kohn et al. [4] corrected their species identification in Kohn and Buhrnheim [48] to *M. opisthonemae* without further explanation.

In our material, the hologenophore possess collapsed and non-collapsed eggs. The collapsed eggs may look as if they bear filaments and spines; however, after detailed examination, we concluded that these structures are absent (Figure 1c). Therefore, we agree with Overstreet [46] who, after re-examining the type material of *M. ecaude*, did not report on filaments or spines on the eggs. Although Overstreet [46] did not observe morphological differences between *M. ecaude* and *M. opisthonemae*, there are morphometric differences that can be noticed (Table 1). Newly collected material of *M. ecaude* from *S. brasiliensis* in the present study was used to provide a detailed description of the species together with novel DNA sequence.

**Table 1:** Comparative metrical data of *Myosaccium* spp.

Species	*Myosaccium ecaude* Montgomery, 1957					*Myosaccium opisthonemae* (Siddiqi & Cable, 1960)		
Source	Present study		Montgomery [38]	León-Règagnon et al. [39]		Overstreet [46]	Siddiqi and Cable [47]	Kohn and Buhruheim [48]
Locality	Atlantic Ocean, Rio de Janeiro, Brazil		Pacific Ocean, California, USA	Pacific Ocean, La Chamela Bay, Mexico		Atlantic Ocean, Florida, USA	Atlantic Ocean, Playa Mani, Puerto Rico	Atlantic Ocean, Rio de Janeiro, Brazil
Host	*Sardinella brasiliensis*		*Sardinops sagax*	*Opisthonema libertae*		*Sardinella aurita*	*Opisthonema oglinum*	*Sardinella aurita*
	Range (*n* = 4)	Mean	Range (*n* = 10)	Range (*n* = 30)	Mean	Range (*n* = 12)	Range (*n* = 40)	Range (*n* = NP)
Body length	924–1009	906	1380–1530	920–1120	1020	440–900	534–827	650–1040
Body width	221–248	232	360–400	280–350	320	–	153–193	120–200
Forebody length	182−224	204	–	–	–	–	–	–
Hindbody length	449−629	538	–	–	–	–	–	–
Pre-oral lobe length	17−26	21	–	–	–	–	–	–
Oral sucker length	71−88	79	90–100	60–80	70	–	52–69	40–80
Oral sucker width	78−102	88	110–120	80–140	90	–	68–79	50–90
Pharynx length	40−51	47	60–70	–	–	–	31–37	30–50
Pharynx width	47−54	50	60–70	–	–	–	–	40–60
Ventral sucker length	126−169	143	–	150–190	170	–	94–120	170–170
Ventral sucker width	140−169	154	–	140–210	180	–	–	70–170
DIBAE	118−168	139	–	–	–	–	–	–
DTVS	44−106	62	–	–	–	–	–	–
Anterior testis length	29−53	40	–	–	–	–	29–69	30–80
Anterior testis width	47−51	49	–	–	–	–	–	20–70
Posterior testis length	31–50	40	–	–	–	–	–	30–90
Posterior testis width	51–67	62	–	–	–	–	–	30–70
Post-testicular region length	322–410	381	–	–	–	–	–	–
Seminal vesicle length	99−152 (*n* = 3)	131	–	–	–	–	–	30–180
Seminal vesicle width	57−74 (*n* = 3)	63	–	–	–	–	–	30–60
Sinus-sac length	73–105	90	130–170	–	–	–	–	60
Sinus-sac width	21–26	24	30–40	–	–	–	–	–
Ovary length	43−66	57	–	–	–	–	39–69	30–50
Ovary width	64−80	69	–	–	–	–	–	20–50
Vitellarium length	81–117	107	–	–	–	–	–	–
Vitellarium width	85–111	101	–	–	–	–	–	–
Egg length	23–30 (*n* = 10) *	28	15–18	26–29	28	21–26	29–32	30–41
Egg width	8–13 (*n* = 10)*	9	9	10–13	11	9–11	12–15	9–13
Body length/body width	1:3.35–4.19	1:3.90	–	–	–	–	–	–
Oral/ventral sucker width	1:1.66−1.79	1:1.75	1:1.57–1.75	1:2.30	1:230	1:1.60–1.80	1:160	1:1.45–2.32
Forebody/body length, %	21−24	23	–	–	–	–	–	–
Post-testicular region/body length, %	39–44	42	–	–	–	–	–	–

* Measurements of collapsed eggs. DIBAE = distance from intestinal bifurcation to anterior extremity, DTVS = distance from testes to ventral sucker, NP = not provided.

**Dinurinae Looss, 1907**
***Ectenurus* Looss, 1907**
***Ectenurus virgula* Linton, 1910**
*Hosts*: *Anisotremus virginicus* (Linnaeus) (Haemulidae), *Decapterus punctatus* (Cuvier) (Carangidae), *Prionotus punctatus* (Bloch) (Triglidae).
*Infection rates*: *A. virginicus*, two out of five; one–five specimens per fish; six specimens in total; *D. punctatus*, one out of one; two specimens in total; *P. punctatus* 1 out of 23; two specimens in total.
*Representatives DNA sequences*: OP918121, OP918122, OP918126 (28); OP918136 (ITS1-5.8S-ITS2); OP948304 (*cox*1).
*Voucher material*: CHIOC 39798; CHIOC 39799 a–e; CHIOC 39800 a,b.

Description (Figure 1d,e). (Based on five paragenophores and one hologenophore; measurements of paragenophores in Table 2 and hologenophore in description): Body elongate, dorso-ventrally flattened. Maximum width at level of ventral sucker or close to posterior body extremity, 361. Tegument covered with conspicuous plications to level of vitellarium. Forebody short, 208. Ecsoma well developed, withdrawn (*n* = 5) or partially extruded (*n* = 1).

Pre-oral lobe distinct, 11 long. Oral sucker muscular, well developed, spherical or subspherical, ventro-subterminal, 104 long, 100 wide. Prepharynx absent. Pharynx muscular, well developed, spherical or subspherical, 65 long, 72 wide. Oesophagus short, 20 long. “Drüsenmagen” present. Intestinal bifurcation just anterior to ventral sucker. Caeca blind, with thick walls and narrow lumen, usually terminates in body or inside of ecsoma when it is extruded (*n* = 1). Ventral sucker muscular, well developed, subspherical, elongate-oval or transversely oval, 300 long, 319 wide, larger than oral sucker (1:3.19), pre-equatorial.

Testes 2, oblique to tandem, contiguous, entire, pre-ovarian, median, in anterior hindbody, separated from ventral sucker (contiguous in hologenophore); subspherical (*n* = 2) to subtriangular (*n* = 4), anterior testis, 132 long, 158 wide, posterior testis, 137 long, 150 wide. Seminal vesicle thin walled, 260 long, 96 wide; tripartite, anterior portion pyriform, 53 long, 44 wide; middle portion subspherical, 71 long, 61 wide; posterior portion elongate-oval, 132 long, 96 wide (Figure 1e). Seminal vesicle in anterior hindbody, connected to pars prostatica by aglandular duct. Pars prostatica very short, tubular, invested by prostatic cells, in anterior hindbody. Ejaculatory vesicle conspicuous, spherical, enclosed within sinus-sac. Sinus-sac elongate, tubular, dorsal to ventral sucker, with muscular wall, 145 long, 22 wide. Hermaphroditic duct straight, enclosed within sinus-sac and sinus-organ. Permanent sinus-organ elongate, tubular and muscular. Genital atrium short. Genital pore median, just anterior to intestinal bifurcation (Figure 1e).

Ovary median (*n* = 3), dextral (*n* = 2) or sinistral (*n* = 1), entire, subtriangular (*n* = 4) to transversely oval (*n* = 2), 71 long, 183 wide, in posterior half of hindbody, contiguous with posterior testis. Vitellarium seven elongate digitiform lobes (three sinistral and four dextral), contiguous with ovary, 243 long, 292 wide. Juel’s organ and Mehlis’ gland not observed. Uterus coiled, restricted to body or extends up to one-fifth length of ecsoma (*n* = 1) when it is extruded. Metraterm not differentiated, terminal part of uterus passes into sinus-sac ventrally, joins male duct forming hermaphroditic duct. Eggs numerous, 17–19 × 9–12 (*n* = 10).

Excretory vesicle not observed; excretory pore terminal. 

***Remarks*:** Specimens obtained in this study agree well with the generic diagnosis of *Ectenurus* Looss, 1907 provided by Gibson [8] in having a plicated body surface, a seminal vesicle divided into three portions in the anterior hindbody, the presence of a sinus-sac, a permanent sinus-organ, and a short pars prostatica connected to the seminal vesicle by a distinct, long, and occasionally convoluted aglandular duct.

Morphologically, the present specimens most closely resemble *E. virgula* described from the stomach of round sardinella, *Sardinella aurita* Valenciennes (=*Clupanodon pseudohispanica*) (type host) in Dry Tortugas, Florida and from *Selar* (=*Trachurops*) *crumenophthalmus* (Bloch) at Woods Hole, Massachusetts, USA, north-western Atlantic Ocean by Linton [49]. Specimens corresponded in body shape, plicated tegument, by possessing an elongate sinus-sac which reaches to the mid-level of the ventral sucker or more posteriorly and not lying in the forebody, a tripartite seminal vesicle, contiguous testes, an ovary contiguous with the posterior testis, and in egg size (16–19 × 8–12 vs. 17–8). However, the maxima for all dimensions was lower in our specimens, except for the sinus-sac length (125–194 vs. 180–260) and eggs (16–19 × 8–12 vs. 17–8). In comparison with specimens collected from *Caranx hippos* (Linnaeus)*, Trachinotus rhodopus* Gill and *Ophioscion scierus* (Jordan and Gilbert) in the Chamela Bay, Jalisco, Mexico, north-eastern Pacific Ocean by León-Règagnon et al. [39], the maxima for all dimensions was lower except for the sucker ratio (1:2.26–3.22 vs. 1:2.3) (Table 2). 

*Ectenurus virgula* parasitises in the stomach of a variety of fish from the Atlantic and Pacific oceans. After the original description, the species was reported parasitising fishes from the Bothidae, Carangidae, Clupeidae, Phycidae, Sciaenidae, Percophidae, and Priacanthidae in the Atlantic Ocean [50,51,52,53,54,55,56,57,58], and from the Carangidae and Sciaenidae in the Pacific Ocean [27].

In Brazil, Travassos et al. [59] reported *E. lepidus* from *Oligoplites saurus* (Bloch and Schneider) in Espírito Santo. However, based on their morphological description, Gibson and Bray [60] claimed that Travassos et al. [59] found *E. virgula*. Thereafter, Pereira et al. [61] reported *E. virgula* from the phycid *U. brasiliensis* (Kaup) in Rio de Janeiro. Our record of *E. virgula* infecting *A. virginicus*, *D. punctatus*, and *Pr. punctatus* collected off the Brazilian coast represent a new host record. Specimens of *E. virgula* collected in our study contributed to a novel detailed description of the species and to generation of DNA sequences.

**Table 2:** Comparative metrical data of *Ectenurus virgula* Linton, 1910.

Source	Present Study		León-Règagnon et al. [39]		Linton [49]
Locality	Atlantic Ocean, Rio de Janeiro, Brazil		Pacific Ocean, Jalisco, Mexico		Atlantic Ocean, Florida, USA
Host	*Anisotremus virginicus*, *Prionotus punctatus*		*Caranx hippos, Ophioscion scierus, Trachinotus rhodopus*		*Sardinella aurita*
	Range (*n* = 5)	Mean	Range (*n* = 9)	Mean	Range (*n* = 1)
Body length	1061–1278	1157	1450–1600	1520	3000
Body width	198–310	253	440–510	470	500
Ecsoma length	252 (*n* = 1)	252	560–880	720	–
Total length	1342 (*n* = 1)	1342	–	–	–
Forebody length	163−239	189	–	–	–
Hindbody length	684−818	743	–	–	–
Pre-oral lobe length	13−19	17	–	–	–
Oral sucker length	74−94	82	150	–	150
Oral sucker width	73−95	82	–	–	–
Pharynx length	43−56	49	–	–	80
Pharynx width	42−54	50	–	–	–
Oesophagus length	20–41	32	–	–	–
Ventral sucker length	186−276	212	320	–	380
Ventral sucker width	172−281	225	–	–	–
DIBAE	112−179	156	–	–	–
DTVS	32−118	66	–	–	–
Anterior testis length	96−171	121	–	–	–
Anterior testis width	91−114	102	–	–	–
Posterior testis length	95–162	114	–	–	–
Posterior testis width	77–120	102	–	–	–
Post-testicular region	412–547	488	–	–	–
Seminal vesicle length	85−183	126	–	–	–
Seminal vesicle width	29−88	53	–	–	–
Sinus-sac length	125–194	154	–	–	180–260*
Sinus-sac width	20–32	27	–	–	–
Ovary length	61−92	79	–	–	–
Ovary width	99−125	111	–	–	–
Vitellarium length	99–151	123	–	–	–
Vitellarium width	149–242	191	–	–	–
Egg length	16–19	17	–	–	17
Egg width	8–12	10	–	–	8
Body length/body width	1:4.61–5.51	1:4.66	–	–	–
Oral/ventral sucker width	1:2.26−3.22	1:2.75	1:2.3	–	–
Ecsoma/body length, %	23 (*n* = 1)	23	–	–	–
Forebody/body length, %	15−20	16	–	–	–
Post-testicular region/body length, %	39–45	42	–	–	–

* Reported by Bray [62] after examination of the type material. DIBAE = distance from intestinal bifurcation to anterior extremity, DTVS = distance from testes to ventral sucker.

**Hemiurinae Looss, 1899**
***Parahemiurus*** **Vaz and Pereira, 1930**
***Parahemiurus merus*** **(Linton, 1910)**
*Hosts*: *Harengula clupeola* (Cuvier), *Sardinella brasiliensis* (Steindachner) (Clupeidae).
*Infection rates*: *H. clupeola*, two out of three; two–six specimens per fish; eight in total; *S. brasiliensis*, one out of two; two specimens in total.
*Representatives DNA sequences*: OP918124, OP918125 (28S).
*Voucher material*: CHIOC 39909; CHIOC 39910 a–e.

**Figure 2:**
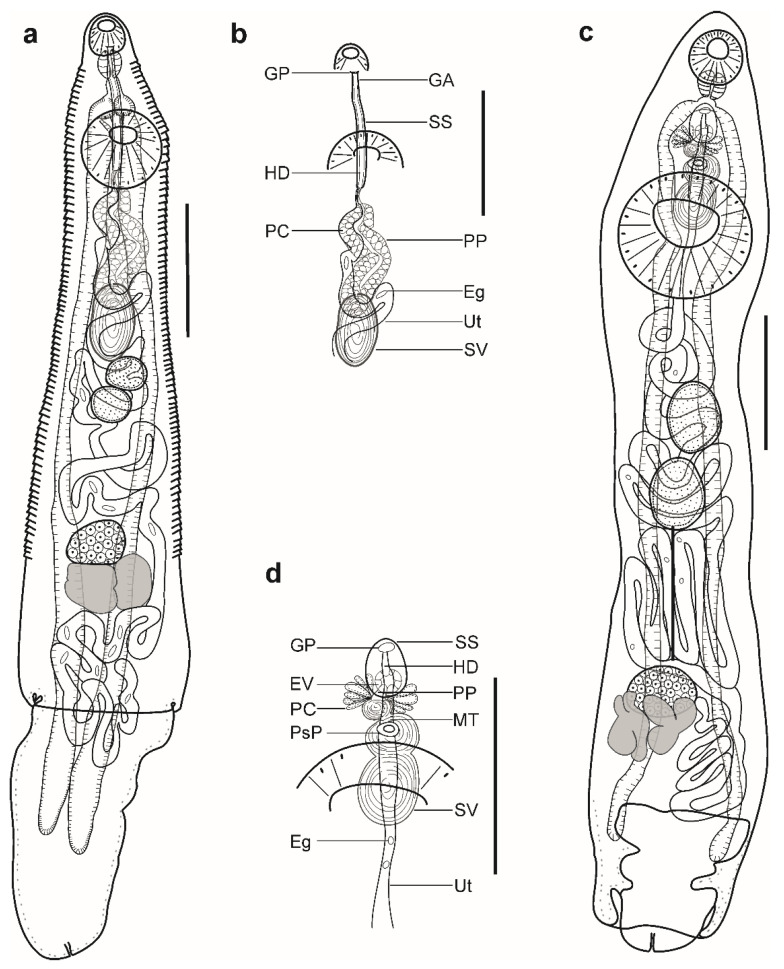
Hemiurid parasites of marine fishes from Brazil. (**a**,**b**) Adult of *Parahemiurus merus* ex *Harengula clupeola*: (**a**) complete specimen, ventral view; (**b**) detail of the terminal genitalia, ventral view. (**c**,**d**) Adult of *Lecithochirium microstomum* ex *Trichiurus lepturus*: **c** complete specimen, ventral view; (**d**) detail of the terminal genitalia, ventral view. *Scale bars* (**a**,**b**): 400 µm, (**c**,**d**): 500 µm. *Abbreviations*: Eg, eggs; EV, ejaculatory vesicle; GA, genital atrium; GP, genital pore; HD, hermaphroditic duct; PC, prostatic cells; PP, pars prostatica; SS, sinus-sac; SV, seminal vesicle; Ut, uterus.

Description (Figure 2a,b). (Based on four paragenophores and two hologenophores; measurements of paragenophores in Table 3 and hologenophores in description): Body elongate, dorso-ventrally flattened, 993–1174 long. Maximum width close to posterior body extremity, 345–393. Tegument covered with conspicuous plications to level of posterior margin of ovary. Forebody short, 138–158, representing 13–14% of body length. Ecsoma well developed, withdrawn (*n* = 2), protruded (*n* = 3) or partially extruded (*n* = 1).

Pre-oral lobe distinct, 15–28 long. Oral sucker muscular, well developed, spherical or subspherical, ventro-subterminal, 80–89 long, 90–100 wide. Prepharynx absent. Pharynx muscular, well developed, spherical or subspherical, 55–56 long, 53–58 wide. Oesophagus short, 22–42 long. “Drüsenmagen” present. Intestinal bifurcation just anterior to ventral sucker. Caeca blind, with thick walls and wide lumen, terminates in body or inside of ecsoma when it is extruded (*n* = 4). Ventral sucker muscular, well developed, subspherical, 172–182 long, 189–194 wide, larger than oral sucker (1:1.94–2.10), pre-equatorial.

Testes 2, oblique to tandem, contiguous, entire, pre-ovarian, median, in middle of hindbody, separated from ventral sucker; anterior testis subspherical, elongate-oval or transversely oval, 81–91 long, 87–120 wide, posterior testis subspherical, elongate-oval or transversely oval, 81–95 long, 113–116 wide. Distance between ventral sucker and anterior testis 114–162. Post-testicular field 511–519, 44–51% of body length. Seminal vesicle thick-walled, saccular, 142–165 long, 67–97 wide. Seminal vesicle in anterior hindbody, connected to pars prostatica by aglandular duct. Pars prostatica long, tubular, convoluted, densely invested by prostatic cells, in anterior hindbody (Figure 2b). Sinus-sac elongate, tubular, muscular, dorsal to ventral sucker, with muscular wall, 178–220 long, 14–16 wide. Hermaphroditic duct straight, enclosed within sinus-sac. Genital atrium short. Genital pore median, ventral to oral sucker.

Ovary median (*n* = 2), dextral (*n* = 3), or sinistral (*n* =1), entire, subspherical or transversely oval, 67–93 long, 111–140 wide, in posterior half of hindbody, always separated from posterior testis by uterine coils. Distance between posterior testis and ovary 95–131. Vitellarium in 2 lateral subsymmetrical oval masses with irregular margins, dextral mass 125–130 long, 88–93 wide, sinistral mass 81–118 long, 77–139 wide. Juel’s organ and Mehlis’ gland not observed. Uterine coils posterior to vitellarium, restricted to body or extends to one-fourth length of ecsoma when it is extruded. Metraterm not differentiated, terminal part of uterus passes into sinus-sac ventrally, joins male duct forming hermaphroditic duct. Eggs numerous, large, 22–26 × 9–10 (*n* = 10).

Excretory vesicle not observed; excretory pore terminal.

***Remarks*:** Specimens found in the present study correspond well to the generic diagnosis of *Parahemiurus* Vaz and Pereira, 1930 provided by Gibson [8] in having a well-developed ecsoma, the absence of an ejaculatory vesicle, in possessing a tubular sinus-sac, a vitellarium composed of two distinct oval masses, a plicated body surface, an oval seminal vesicle with a muscular wall, and a tubular pars prostatica. 

Our specimens correspond in their morphology to *Parahemiurus merus* (Linton, 1910) described as *Hemiurus merus* from the stomach of *Sardinella aurita* (=*Clupanodon pseudohispaniciis*) in Tortugas, Florida, USA, north-western Atlantic Ocean by Linton [49]. Specimens corresponded in body shape, having plicated body surface to level of the vitellarium, in possessing a narrow and elongate sinus-sac, an oval seminal vesicle, and contiguous testes. However, except for the egg size (22–27 × 7–11 vs. 27 × 10) (Table 3), the maxima for all dimensions in our specimens was lower. The features and dimensions of our specimens overlap with specimens collected from *Sardinella aurita* Valenciennes in São Paulo, Brazil, specimens collected from *Engraulis anchoita* Hubbs and Marini off the Argentinean and Uruguayan coasts, southwestern Atlantic Ocean by Vaz and Pereira [63] and Timi et al. [64], specimens collected from several fish hosts of the families Carangidae, Clupeidae, Haemulidae, Merlucciidae, Pomatomidae, Salmonidae, Scorpaenidae, and Sparidae in different localities from the Atlantic, Indian, and Pacific oceans in a review published by Bray [65], and specimens collected from several fish hosts of the families Balistidae, Clupeidae, Engraulidae, and Haemulidae in Chamela Bay, Jalisco, Mexico, north-eastern Pacific Ocean by León-Règagnon et al. [39] (Table 3).

*Parahemiurus merus* is reported mainly from clupeid, carangid, salmonid, and engraulid fishes from the Atlantic, Indian, and Pacific oceans [65]. In Brazil, this species has been reported several times in fishes from at least 12 families (Belonidae, Carangidae, Clupeidae, Dactylopteridae, Engraulidae, Haemulidae, Ophidiidae, Phycidae, Pinguipedidae, Pomatomidae, Sciaenidae, and Sparidae) [9]. Wallet and Kohn [66] and Luque et al. [31] reported *P. merus* in *H. clupeola*, and Luque et al. [44] and Moreira et al. [45] reported it in *S. brasiliensis*. Benicio et al. [67] reported it in *Cetengraulis edentulus* (Cubier) in the same region as in our study. Newly collected specimens of *P. merus* have allowed us to provide a detailed description of the species and generate the first DNA sequence data.

**Lecithochirinae Lühe, 1901**
***Lecithochirium floridense*** **(Manter, 1934)**
*Host*: *Percophis brasiliensis* Quoy and Gaimard (Percophidae).
*Infection rates*: one out of five; five specimens in total.
*Representatives DNA sequences*: OP918131 (28S); OP918025 (*cox*1).
*Voucher material*: CHIOC 39902 a–c.

***Remarks*:** Specimens found in the present study correspond well to the generic diagnosis of *Lecithochirium* Luhe, 1901 provided by Gibson and Bray [60] and Gibson [8] in having a well-developed ecsoma, a pre-oral lobe, a tubular pars prostatica, a vitellarium of two lateral masses divided into three and four short lobes, and eggs without polar filaments. Comparative sequence analyses (see below) demonstrated that the 28S rDNA and *cox*1 sequences of our isolate from the percophid *P. brasiliensis* identified as *L. floridense* was identical to sequences of the same species reported in *Auxis thazard* (Lacépède) in Rio de Janeiro, Brazil by Pantoja et al. [7].

*Lecithochirium floridense* is a parasite of the stomach of a variety of marine fish species. To date, *L. floridense* has been reported from fishes from at least 17 families, including the present data, with the majority of records coming from the western Atlantic Ocean (Pantoja et al. [7]). In Brazil, *L. floridense* was reported from *A. thazard* in Rio de Janeiro, southwestern Atlantic Ocean. Our record of *L. floridense* infecting *Pe. brasiliensis* collected off the Brazilian coast represents a new host record for this species. We do not provide a description of the specimens of this species because a detailed morphological description of this species with DNA sequence data was provided by Pantoja et al. [7].

***Lecithochirium microstomum* Chandler, 1935**
*Hosts*: *Prionotus punctatus* (Bloch) (Triglidae), *Trichiurus lepturus* (Linnaeus) (Trichiuridae).
*Infection rates*: *Pr. punctatus*, 1 out of 23; three specimens in total; *T. lepturus*, one out of one; 10 specimens in total.
*Representative DNA sequences*: *L. microstomum*: OP918119, OP918120, OP918127 (28S); OP918137 (ITS2); OP918021, OP948302, OP918022 (*cox*1); *T. lepturus*: OP905634 (*cox*1).
*Voucher material*: CHIOC 39903 a,b; CHIOC 39904 a–g.

Description (Figure 2c,d). (Based on six paragenophores and one hologenophore; measurements of paragenophores in Table 4 and hologenophores in description): Body elongate, dorso-ventrally flattened. Maximum width at ventral sucker level, 679. Tegument smooth. Forebody short, 574. Ecsoma well developed, withdrawn (*n* = 5) or protruded (*n* = 2).

Pre-oral lobe distinct, 36 long. Oral sucker muscular, well developed, spherical or subspherical, ventro-subterminal, 194 long, 176 wide. Prepharynx absent. Pharynx muscular, well developed, spherical or subspherical, 112 long, 104 wide. Oesophagus, 80 long (*n* = 4). “Drüsenmagen” present. Presomatic pit non-aglandular anterior to ventral sucker (Figure 2c,d). Intestinal bifurcation in anterior forebody. Caeca blind, with thick walls and narrow lumen, usually terminates in body or inside ecsoma when extruded (*n* = 2). Ventral sucker muscular, well developed, subspherical, transversely oval or elongate-oval 564 long, 527 wide, larger than oral sucker (1:2.99), pre-equatorial.

Testes 2, obliquely tandem, contiguous or separated, entire, pre-ovarian, median, in anterior hindbody, separated from ventral sucker; anterior testis elongate-oval, 298 long, 231 wide, posterior testis elongate-oval, 293 long, 224 wide. Distance between ventral sucker and anterior testis 254. Seminal vesicle thin walled, 395 long, 184 wide, tripartite; anterior portion subspherical, 52 long, 48 wide, middle portion transversely oval, 102 long, 145 wide, posterior portion elongate-oval, 241 long, 184 wide (Figure 2d). Seminal vesicle between genital pore and middle level of ventral sucker, dorsal to ventral sucker, connected to pars prostatica by aglandular duct. Pars prostatica short, tubular, invested by prostatic cells, in mid-forebody (Figure 2d). Ejaculatory vesicle conspicuous, spherical, enclosed within sinus-sac. Sinus-sac large, elongate-oval, between pharynx and presomatic pit, with muscular wall, 228 long, 171 wide. Hermaphroditic duct enclosed within sinus-sac, short, straight, opens directly through genital pore. Genital pore median, at level of intestinal bifurcation or slightly posterior to it. 

Ovary median (*n* = 3), dextral (*n* = 3) or sinistral (*n* = 1), spherical, subspherical, elongate-oval or transversely oval, entire, 231 long, 316 wide, in posterior half of hindbody, always separated from posterior testis by uterine coils, posterior part dorsal to vitellarium. Distance between posterior testis and ovary 542. Vitellarium in two lateral compact masses, divided into three and four digitiform lobes, 329 long, 516 wide. Juel’s organ and Mehlis’ gland not observed. Uterus coiled, restricted to body or extends to 15% of length of ecsoma (*n* = 1). Metraterm passes into sinus-sac ventrally, joins male duct just distally to ejaculatory vesicle forming hermaphroditic duct. Eggs numerous, 16–20 × 10–12 (*n* = 10).

Excretory vesicle not observed; excretory pore terminal.

***Remarks*:** Specimens found in the present study correspond well to the generic diagnosis of *Lecithochirium* in characters as mentioned above. Following the key to the species-group of *Lecithochirium* proposed by Bray [68], our specimens belong to the ‘Microstomum-group’. This diagnosis is based on the presence of a non-glandular presomatic pit, a vitellarium of compact masses with distinct and digitiform lobes, terminal genitalia of the ‘musculus’ type, a non-muscular seminal vesicle, and the absence of internal elevations of the ventral sucker.

In comparison to species from the ‘Microstomum-group’, our specimens can be distinguished from *L. alectis* in the position of the seminal vesicle (antero-dorsal to the ventral sucker vs. entirely anterior to the ventral sucker), from *L. mecosaccum, L. antennari, L. chaetodontis*, and *L. maomao* in the position of the testes (obliquely tandem vs. obliquely symmetrical) and from *L. priacanthi* in the form of a preacetabular pit (poorly developed vs. well developed into typical sucker).

Morphologically, our material closely resembles *L. manteri* Teixeira de Freitas and Gomes, 1971 and *L. microstomum* Chandler, 1935, in possessing oblique tandem testes separated from the ventral sucker and a similar ratio of the suckers (1:2.53–3.12 vs. 1:2.81–3.37 vs. 1:2.5–2.8, respectively). However, our material can be distinguished from *L. manteri* of Teixeira de Freitas and Gomes [69] in possessing a narrower body (641–769 vs. 1040–1310), a smaller pharynx (95–106 × 93–111 vs. 110–120 × 120–130), in partition of seminal vesicle (tripartite vs. bipartite), and a smaller ovary (184–271 × 239–292 vs. 330–420 × 350–430).

Our specimens most closely resemble *L. microstomum* from the Largehead hairtail, *T. lepturus* collected in Galveston Bay, Texas, USA particularly in body length (3104–4292 vs. 2750–4800) and by the presence of an oesophagus and tripartite seminal vesicle. However, our specimens differ from the material of Chandler [70] by possessing a narrower body (641–769 vs. 875–1000) and more elongate eggs (18–26 × 10–15 vs. 16–12). The dimensions of our material fall within those of Teixeira de Freitas and Kohn [71], except for the posterior testis width (187–242 vs. 250–450), the seminal vesicle length (188–462 vs. 670–900), and the ovary length (239–292 vs. 330–430). In comparison with the material of Wallet and Kohn [66], our specimens differ as they possess a more elongate oral sucker (170–198 vs. 140–150), a shorter pharynx (95–106 vs. 190–210), a more elongated anterior testis (244–302 vs. 180–230), and a more elongated ovary (184–271 vs. 150–180). Morphology and dimensions of our specimens overlap with specimens collected from fish hosts of the families Carangidae, Engraulidae, Fistularidae, Lutjanidae, and Scombridae in Chamela Bay, Jalisco, Mexico, northeastern Pacific Ocean by León-Règagnon et al. [39]. In comparison with material collected from *E. anchoita* off the Argentinean and Uruguayan coasts, southwestern Atlantic Ocean by Timi et al. [64], the maxima for body and most internal organs of our specimens are much higher, except for the seminal vesicle width (65–236 vs. 25–74) and the egg size (18–26 × 10–15 vs. 15–19 × 8–11) for which the dimensions overlap.

*Lecithochirium microstomum* is a parasite of a variety of marine fishes. To date, this species has been reported in fishes belonging to at least 26 families (Anguillidae, Ariidae, Batrachoididae, Bothidae, Carangidae, Centropomidae, Coryphaenidae, Engraulidae, Fistulariidae, Gempylidae, Gerreidae, Labridae, Lutjanidae, Malacanthidae, Paralichthyidae, Percophidae, Phycidae, Pinguipedidae, Pomatomidae, Rachycentridae, Sciaenidae, Scombridae, Serranidae, Sparidae, Synodontidae, and Trichiuridae), with the majority of records coming from the western Atlantic Ocean and eastern Pacific Ocean [9,39,72,73,74]. Razarihelisoa [75] reported this species from *Bothus pantherinus* (Rüppell) in Nosy Be, Madagascar, southwestern Indian Ocean. Kardousha [76] reported this species from *Euthynnus affinis* (Cantor) in the Arabian Gulf, northwestern Indian Ocean.

In Brazil, *L. microstomum* has been reported from fishes of 11 families (Carangidae, Engraulidae, Gempylidae, Gerreidae, Paralichthyidae, Percophidae, Phycidae, Pinguipedidae, Sciaenidae, Scombridae, and Serranidae) [9,67]. Teixeira de Freitas and Kohn [71], Wallet and Kohn [66], Silva et al. [77,78], and Carvalho and Luque [79] reported *L. microstomum* from the same fish host, *T. lepturus*, and the same locality as in the present study. Newly collected material of *L. microstomum* in the present study has allowed us to provide a detailed description of the species together with DNA sequence data.

***Lecithochirium*** **cf. *muraenae* Manter, 1940**
*Host: Gymnothorax vicinus* (Castelnau) (Muraenidae).
*Infection rates*: one out of two; four specimens in total.
*Representative DNA sequences*: OP918128 (28S); OP918138 (ITS2); OP918023, OP948305 (*cox*1).
*Voucher material*: CHIOC 39901 a–d.

**Figure 3:**
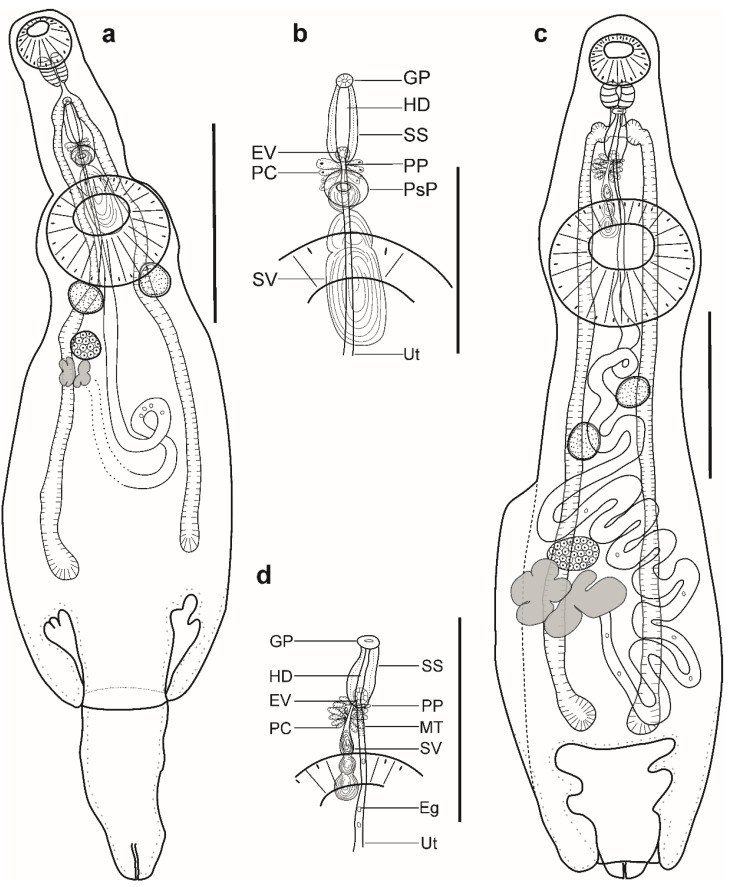
Hemiurid parasites of marine fishes from Brazil. (**a**,**b**) Adult of *Lecithochirium* cf. *muraenae ex Gymnothorax vicinus*: (**a**) complete specimen, ventral view; (**b**) detail of the terminal genitalia, ventral view. (**c**,**d**) Adult of *Lecithochirium* sp. ex *Trichiurus lepturus*: (**a**) complete specimen, ventral view; (**b**) detail of the terminal genitalia, ventral view. *Scale bars* (**a**): 700 µm, (**b**): 350 µm, (**c**,**d**): **500***. Abbreviations*: Eg, eggs; EV, ejaculatory vesicle; GP, genital pore; HD, hermaphroditic duct; PC, prostatic cells; PP, pars prostatica; SS, sinus-sac; SV, seminal vesicle; Ut, uterus.

Description (Figure 3a,b). (Based on three paragenophores and one hologenophore; measurements of paragenophores in Table 4 and hologenophore in description; Figure 3a): Body small, plump, elongate, pyriform. Maximum width at middle of hindbody, 912. Tegument smooth. Forebody short 303. Ecsoma well developed, withdrawn or protruded.

Pre-oral lobe short, 13 long. Oral sucker muscular, well developed, subspherical or transversely oval, ventro-subterminal, 160 long, 195 wide. Prepharynx absent. Pharynx muscular, well developed, subspherical or elongate-oval. Oesophagus short, 20 long. Presomatic pit present. Caeca blind, with thick walls and narrow lumen, terminates in body. Ventral sucker muscular, well developed, subspherical or transversely oval, 402 long, 439 wide, larger than oral sucker (1:2.25), pre-equatorial.

Testes 2, symmetrical, separated, entire, pre-ovarian, in anterior hindbody, contiguous with ventral sucker; dextral testis subspherical or transversely oval, 115 long, 161 wide, sinistral testis subspherical or transversely oval, 155 long, 158 wide. Seminal vesicle thin walled, tripartite, anterior and median portions subspherical, posterior portion elongate-oval (Figure 3d). Seminal vesicle between presomatic pit and middle level of ventral sucker, antero-dorsal to ventral sucker, connected to pars prostatica by aglandular duct. Pars prostatica short, tubular, invested by prostatic cells, anterior to ventral sucker (Figure 3b). Ejaculatory vesicle conspicuous, spherical, enclosed within sinus-sac. Sinus-sac elongate-oval, posterior to intestinal bifurcation, with muscular wall. Hermaphroditic duct enclosed within sinus-sac, straight, opens directly through the genital pore. Genital pore median at level of intestinal bifurcation.

Ovary dextral (*n* = 2) or sinistral (*n* = 2), entire, transversely oval, 128 long, 172 wide, in anterior half of hindbody, always separated from testes, contiguous with vitellarium. Distance between testes and ovary 63. Vitellarium in two lateral compact masses, divided into three and four short lobes, 205 long, 154 wide. Juel’s organ and Mehlis’ gland not observed. Empty portion of uterus were not observed because of thin uterine wall. Metraterm not differentiated. Eggs small, 10–13 × 9–12 (*n* = 10).

Excretory vesicle not observed; excretory pore terminal.

***Remarks*:** Specimens collected in this study possess features that fully correspond to the generic diagnosis of the genus *Lecithochirium* in characters as mentioned above. According to the key to the species-group of *Lecithochirium* proposed by Bray [68], our specimens belong to the ‘Microstomum-group’ based on the presence of a non-glandular presomatic pit, the absence of internal elevations in the ventral sucker, a non-muscular seminal vesicle, terminal genitalia of the ‘musculus’ type, and a vitellarium represented by compact masses with distinct and digitiform lobes.

Comparing to the species in the ‘Microstomum-group’, our specimens could be distinguished from *L. alectis* in the position of the seminal vesicle (antero-dorsal to the ventral sucker vs. entirely anterior to the ventral sucker), from *L. mecosaccum* in possessing a short sinus-sac (116–129 vs. 170–300), and from *L. antennari, L. chaetodontis*, *L. maomao, L. microstomum*, and *L. priacanthi* in the position of the testes (obliquely symmetrical vs. obliquely tandem).

Our specimen is morphologically similar to *L. albulae* Yamaguti, 1970 described from the bonefish *Albula vulpes* (Linnaeus) collected in Hawaii, USA, particularly as they possess obliquely symmetrical testes, a tripartite seminal vesicle, a seminal vesicle posterodorsal to the ventral sucker, and similar ratios of suckers (1:2.32–2.47 vs. 1:2.1–2.35). However, they differ in the length of the eggs (10–14 × 9–13 vs. 14–23 × 9–13) and in the form of a preacetabular pit (poorly developed vs. well developed into typical sucker).

Morphologically, our material is most similar to the species of *L. muraenae* described by Manter [80], from the stomach of the Hourglass moray *Muraena clepsydra* Gilbert in Cape Elena, Ecuador, southeastern Pacific Ocean, in that they possess a conspicuous and non-glandular presomatic pit, symmetrical testes, a tripartite seminal vesicle, and a vitellarium consisting of two lateral masses divided into three and four short lobes. Nonetheless, specimens in our study differ from the material of Manter [80] by having smaller dimensions for all internal organs (Table 5), in the position of the genital pore (at level of intestinal bifurcation vs. posterior to intestinal bifurcation), and in possessing a straight instead of convoluted hermaphroditic duct. The variation in metrical data, in our opinion, relates to the differences in the fixation method (heat-killed fixation of our material vs. fixation under a cover glass, with the application of slight pressure of the material in Manter [80]). Considering the similarities in morphology and group of hosts (muraenids), we provisionally identify our specimens as *L. muraenae*.

*Lecithochirium muraenae* is a parasite of muraenid fishes. After the original description, the species was reported in *Gymnothorax porphyreus* Guichenot (=*G. wieneri*) in El Callao, Peru, southeastern Pacific Ocean [81]. Our record of *L. muraenae* infecting *G. vicinus* off the Brazilian coast potentially represents a new host and geographical record for this species. Additionally, we provide DNA sequence data that may further help to elucidate the taxonomic identity of our specimens.

***Lecithochirium synodi* Manter, 1931**
*Hosts*: *Anisotremus virginicus* (Linnaeus) (Haemulidae), *Pseudopercis numida* Miranda Ribeiro (Pinguipedidae).
*Infection rates*: *A. virginicus*, one out of five; four specimens in total; *Ps. numida*, one out of one; three specimens in total.
*Representative DNA sequences*: OP918129, OP918130, OP918132 (28S); OP918139 (ITS2); OP918024, OP918026 (*cox*1); *Ps. numida*: OP925860 (*cox*1).
*Voucher material*: CHIOC 39906 a,b; CHIOC 39907 a,b.

***Remarks*:** Specimens found in the present study correspond well to the generic diagnosis of *Lecithochirium* in characters as mentioned above. Our specimens collected from the haemulid *A. virginicus* and from the pinguipedid *Ps. numida* are morphologically similar to those described from *A. thazard* by Pantoja et al. [7] in Rio de Janeiro, Brazil. The identification of our specimens as *L. synodi* was confirmed via comparative sequence analyses, which demonstrated that the 28S rDNA sequence of our isolate of *Lecithochirium* was identical to two isolates of *L. synodi* reported by Pantoja et al. [7]. Their *cox*1 sequences differed by 0.23–0.45% (1–2 nt).

*Lecithochirium synodi* is a parasite of the stomach of a variety of marine fish species. To date, this species has been reported from fishes from at least six families (Haemulidae, Monocanthidae, Paralichthyidae, Pinguipedidae, Scombridae, and Synodontidae), including the present data, with the majority of records coming from the western Atlantic Ocean [7]. Our record of *L. synodi* infecting *A. virginicus* and *Ps. numida* collected off the Brazilian coast represents two new host records for this species. We do not provide the description of the specimens of this species because a detailed morphological description of this species with DNA sequence data was recently provided by Pantoja et al. [7].

***Lecithochirium* sp.**
*Host*: *Trichiurus lepturus* (Linnaeus) (Trichiuridae).
*Infection rates*: one out of one; three specimens in total.
*Representative DNA sequences*: not available.
*Voucher material*: CHIOC 39905.

Description (Figure 3c,d). (Based on one specimen; measurements in Table 5): Body elongate, dorso-ventrally flattened. Maximum width close to posterior body extremity. Tegument smooth. Forebody short. Ecsoma well developed, withdrawn.

Pre-oral lobe distinct. Oral sucker muscular, well developed, spherical, ventro-subterminal. Prepharynx absent. Pharynx muscular, well developed, subspherical. Oesophagus short. “Drüsenmagen” present. Presomatic pit absent. Intestinal bifurcation in anterior forebody. Caeca blind, with thick walls and narrow lumen, terminates close to posterior extremity of body. Ventral sucker muscular, well developed, subspherical, larger than oral sucker, pre-equatorial.

Testes 2, oblique, separated, entire, pre-ovarian, in anterior hindbody, separated from ventral sucker; dextral testis elongate-oval, sinistral testis subspherical. Post-testicular field almost half of body length. Seminal vesicle thin walled, tripartite; anterior portion pyriform; median and posterior portions subspherical (Figure 3d). Seminal vesicle between intestinal bifurcation and middle level of ventral sucker, antero-dorsal to ventral sucker, connected to pars prostatica by aglandular duct. Pars prostatica short, tubular, invested by prostatic cells, anterior to ventral sucker (Figure 3b). Ejaculatory vesicle conspicuous, spherical, enclosed within sinus-sac. Sinus-sac large, elongate-oval, with muscular wall, at level of intestinal bifurcation. Hermaphroditic duct enclosed within sinus-sac, straight, opens directly through the genital pore. Genital pore just posterior to pharynx.

Ovary dextral, entire, transversely oval, in posterior half of hindbody, always separated from posterior testis by uterine coils, contiguous with vitellarium. Vitellarium in two lateral compact masses, divided into three and four short lobes. Juel’s organ and Mehlis’ gland not observed. Uterus coiled, restricted to body. Metraterm passes into sinus-sac ventrally, joins male duct just distally to ejaculatory vesicle forming hermaphroditic duct. Eggs numerous, small.

Excretory vesicle not observed; excretory pore terminal. 

***Remarks*:** The specimen found in the present study correspond well to the generic diagnosis of *Lecithochirium* in characters as mentioned above. Following the key to the species-group of *Lecithochirium* proposed by Bray [68], our specimen belongs to the “Musculus-group” based on the absence of a presomatic pit, the presence of a vitellarium comprised of compact masses with distinct and short digitiform lobes, a terminal genitalia of the “musculus” type, a non-muscular seminal vesicle, and the absence of internal elevations in the ventral sucker.

In comparison to species from the “Musculus-group”, our specimen can be distinguished from all other species; from *L. brevicirrus* (Nicoll, 1915), *L. floridense* (Manter, 1934), *L. medius* Acena, 1941, *L. microcercus* (Manter, 1947) and *L. musculus* (Looss, 1907) in the position of the testes (not contiguous with the ventral sucker vs. contiguous or just posterior to the ventral sucker or overlapping it), from *L. imocavus* by having a distinctly wider body (694 vs. 250–400) and from *L. monticellii* (Linton, 1898) in the position of the testes (contiguous vs. separated by uterine coils).

Our single specimen closely resembles *L. monticellii* collected from the stomach of *T. lepturus* in the Atlantic Ocean, Rio de Janeiro, Brazil, by França et al. [15], in that it possesses a genital pore at or slightly posterior to the pharynx, a tripartite seminal vesicle, and testes being separated from the ventral sucker. In comparison with large specimens collected by França et al. [15], our specimen differs by having a wider body (694 vs. 220–520), a longer sinus-sac (179 vs. 50–100), a slightly larger sucker ratio (1:2.46 vs. 1:1.95–2.41), and shorter eggs (11–15 × 9–11 vs. 23–28 × 9–12). In comparison with small specimens collected by França et al. [15], our specimen differs by having a longer body (2607 vs. 1020–1900), a wider body (694 vs. 220–500), a longer seminal vesicle (217 vs. 50–170), a longer sinus-sac (179 vs. 20–100), and shorter eggs (11–15 × 9–11 vs. 15–28 × 10–13) (Table 5). Although our material most resembles the material of França et al. [15], the species identification provided by these authors should be interpreted with caution. Their specimens differ from the type material of *L. monticellii* described by Linton [82], specifically regarding the position of the testes (separated vs. contiguous in Linton [82]).

Out of the “Musculus-group”, our specimen closely resembles *L. trichiuri* (‘Keokeo-group’) described from the same host, *T. lepturus* (=*T. haumela*) in the China Sea by Gu and Shen [83]; it is similar in body shape, it possesses testes separated from the ventral sucker, and has a similar ratio of the suckers (1:2.46 vs. 1:2.4–2.9). However, our specimen lacks a presomatic pit, whereas *L. trichiuri* possess a presomatic pit.

Due to the presence of a single specimen in our material and difficulties in assigning this specimen to any known species, we identify it to the genus level as *Lecithochirium* sp.

### 3.2. Molecular Results

**Figure 4:**
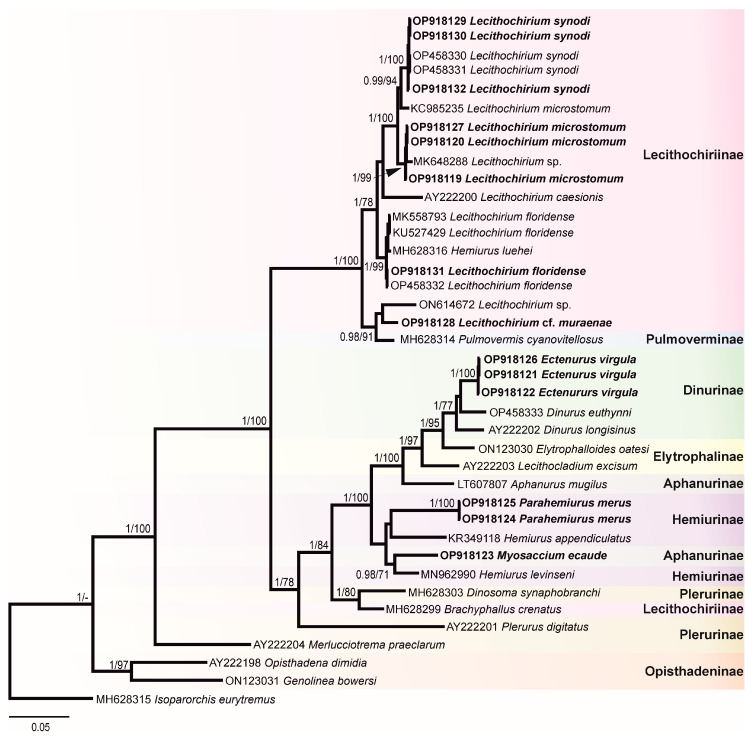
Phylogenetic tree resulted from Bayesian inference (BI) analysis of the 28S rDNA sequences datasets of the Hemiuridae with nodal support values shown at the node as BI/ML (maximum likelihood). Support values <0.90 (BI) and 70 (ML) are not shown. Sequences generated in the present study are highlighted in bold.

In this study, 30 novel sequences were generated for 16 isolates. The phylogenetic relationships of the studied species of the Hemiuridae were assessed based on the partial 28S rDNA sequences. Sequences of ITS1-5.8S-ITS2, ITS2, and *cox*1 were used to calculate the pairwise genetic distances between species and to contribute to a growing DNA sequence library for the Hemiuridae. The phylogenetic tree obtained from the BI analysis based on Alignment 1 (28S rDNA; 1071 nt) is presented in Figure 4. Pairwise genetic distances of this dataset are presented in Table S1. Novel 28S rDNA sequences (*n* = 14) of seven species were positioned in different clades with the members of the family Hemiuridae.

The most surprising result of our molecular analyses is that a sequence of *M. ecaude* (OP918123), a member of the subfamily Aphanurinae according to classifications based on morphology, clustered with a sequence of *Hemiurus levinseni* (MN962990) (a member of the Hemiurinae) collected from *Cylichna alba* (Brown) in White Sea, Russia with strong support. Two identical sequences of *P. merus* generated in the present study (members of the Hemiurinae) and a sequence of *Hemiurus appendiculatus* (Rudolphi, 1802) clustered in the same clade, albeit with low support. The sequence divergence between *M. ecaude* and *He. levinseni*, *He. appendiculatus*, and *P. merus* was 4.84–9.12% (50–79 nt). In comparison with *Aphanurus mugilus* Tang, 1981 (LT607807), a species of the type genus of the Aphanurinae, our sequence of *M. ecaude* differed by 8.32% (86 nt). This result suggests that the position of *M. ecaude* within the Aphanurinae requires revaluation.

Our molecular phylogenetic analyses confirmed the positions of *E. virgula* within the subfamily Dinurinae and *Lecithochirium* spp. within the subfamily Lecithochiriinae. The three sequences of *E. virgula* (OP918121, OP918122, and OP918126) were identical and clustered with sequences of members of the same subfamily Dinurinae, *Di. euthynni* (OP458333) collected from *A. thazard* in Rio de Janeiro, Brazil, and *Di. longisinus* (AY222202) collected from *Coryphaena hippurus* Linnaeus, Jamaica. The sequence divergence between these three species ranged from 3.21 to 3.80% (33–49 nt).

All sequences generated for species of *Lecithochirium* in this study clustered in a strongly supported clade. The three novel sequences of *L. synodi* (GenBank OP918129, OP918130, and OP918132) clustered with sequences of two isolates of the same species (OP458330 and OP458331) found in *A. thazard* in Rio de Janeiro, Brazil and *L. microstomum* Chandler, 1935 (KC985235) collected from *T. lepturus* in the USA. All five sequences of *L. synodi* were identical. The three novel sequences of *L. microstomum* (OP918119, OP918120, and OP918127) were identical and clustered with *Lecithochirium* sp. (MK648288) collected from the same host, *T. lepturus* in Veracruz, Mexico in the same strongly supported subclade. The sequence divergence between our isolates and the isolate from Mexico was 0.47% (5 nt). The three identical novel sequences of *L. microstomum* (OP918119, OP918120, and OP918127) differed from *L. microstomum* (KC985235) by 1.5% (16 nt). The novel sequence of *L. floridense* (OP918131) from *Pe. brasiliensis* grouped with sequences of three isolates of the same species (Figure 4) previously reported from *S. papillosum* in Yucatan Shelf, Mexico (MK558793), in *Pterois volitans* from Northern Gulf of Mexico, USA (KU527429) and in A. thazard from Rio de Janeiro, Brazil (OP458332) and with the sequence of *He. luehei* (MH628316). Krupenko et al. [84] reported that the sequence named as *He. luehei* and published by Sokolov [12] was misidentified. The position of the sequence of *He. luehei* in this clade was also discussed in Pantoja et al. [7]. The intraspecific divergence between five isolates was 0–0.21% (0–2 nt).

Sequences of *Lecithochirium* cf. *muraenae* collected from *G. vicinus* clustered with the sequence of an unidentified species of *Lecithochirium* collected from *Octopus bimaculatus* Verrill, 1883 from Baja California, Mexico, albeit with low support. The third sequence in the same clade was of *Pulmovermis cyanovitellosus* Coil & Kuntz, 1960 (MH628314) collected from *Laticauda semifasciata* (Reinwardt) in Ishigaki Island, Japan; the sequence divergence within this clade was 2.81–4.02% (30–43 nt) with sequences of *Lecithochirium* cf. *muraenae* and *Pu. cyanovitellosus* showing the lowest sequence divergence. The interspecific divergence within the clade containing *Lecithochirium* spp. was 1.21–6.82% (13–73 nt), with *Lecithochirium microstomum* and *L. synodi* demonstrating the lowest interspecific divergence, and *L. caesionis* and *Lecithochirium* sp. (ON614672) demonstrating the highest interspecific divergence.

Pairwise genetic distances of Alignment 2 containing ITS2 sequences (416 nt) *Lecithochirium* spp. is presented in Table S2. The ITS2 sequences of *L. synodi* from *A. virginicus* (OP918139) and *A. thazard* (OP458331) were identical. The interspecific divergence within *Lecithochirium* spp. available for comparison was 1.92–24.33% (8–100 nt), with *L. microstomum* and *L. synodi* exhibiting the lowest interspecific divergence, and *L. synodi* and *L*. cf. *muraenae* exhibiting the highest interspecific divergence. The ITS2 sequences of *E. virgula* (OP918136) and *Di. euthynni* (OP458333) from *A. thazard* in Rio de Janeiro, Brazil (both from the subfamily Dinurinae) differed by 2.97% (13 nt).

Pairwise genetic distances of Alignment 3 containing *cox*1 sequences (443 nt) of *Lecithochirium* spp. is presented in Table S3. The intraspecific divergence of *L. synodi* collected from *A. virginicus* (OP918024), *Ps. numida* (OP918026), and *A. thazard* (OP418194) was 0.23–0.45 (1–2 nt). The intraspecific divergence of *L. microstomum* from *T. lepturus* (OP918021) and *Pr. punctatus* (OP918022) was 1.13% (5 nt). The sequences of *L. floridense* from *P. brasiliensis* (OP918025) and *A. thazard* (OP418195) were identical. The interspecific divergence between *Lecithochirium* spp. available for comparison was 14.9–26% (66–115 nt), with *L*. cf. *muraenae* exhibiting the highest interspecific divergence from other *Lecithochirium* spp.

## 4. Discussion

Following combined morphological and molecular analyses, we report on eight hemiurid species belonging to four genera, namely: *Ectenurus virgula*, *L. floridense*, *L. microstomum*, *Lecithochirium cf. muraenae*, *L. synodi*, *Lecithochirium* sp., *Myosaccium ecaude*, and *Parahemiurus merus*. Our results add new data on the geographical distribution, host associations, and the first DNA sequence data for five hemiurid species.

According to the World Register of Marine Species [85], the genus *Ectenurus* is currently represented by 28 species globally. In Brazil, only two species have been reported, *E. virgula* and *E. yamagutii*, by Nahhas and Powell, 1971 [9] from fishes of three families, Carangidae, Haemulidae, and Phycidae. *Ectenurus virgula* was previously reported in the carangid *O. saurus* and in the phycid *Urophycis brasiliensis* (Kaup) [59,61]. We found this species in taxonomically different hosts, the carangid *D. punctatus*, the haemulid *A. virginicus*, and the triglid *Pr. punctatus*, and confirmed *E. virgula* to be an euryxenous parasite. The relative position of *E. virgula* to *Di. euthynni* and *Di. longisinus* in our phylogenetic analysis (Figure 4) confirmed its taxonomic position within the subfamily Dinurinae. Notably, the DNA sequence divergence between *E. virgula* and *Di. euthynni* was lower than the divergence between congeneric *Di. euthynni* and *Di. longisinus* (3.27%, 34 nt vs. 3.80%, 39 nt, respectively). Morphologically, members of both genera are similar in that they have a plicated body surface, a partitioned seminal vesicle, and the presence of a sinus-organ. However, they differ in the length of the pars prostatica.

*Lecithochirium* is one of the most speciose genera of the family Hemiuridae. The species recognition of the genus, in our view, is often problematic due to the poor descriptions for many species and the lack of critical differentiation analysis at the time of description. Thus, the true species composition of this genus can only be assessed by new, accurate, and precise work, including detailed morphological descriptions together with the incorporation of DNA sequence data. In the present study, the genus *Lecithochirium* is the richest and is represented by five species. Two of these, *L. floridense* and *L. synodi*, were recently reported from *A. thazard* in Brazil with a morphological description and DNA sequences provided [7]. Therefore, the identification of these species was based on both morphological and comparative sequence analyses. Our findings suggest that the host spectrum for both species is wider. Although *L. floridense* is reported for the first time in a percophid fish and *L. synodi* in haemulid and pinguipedid fishes, the euroxenous nature of both species was already observed in previous studies [7,86].

For the three remaining species of *Lecithochirium,* we provided detailed morphological descriptions, with two of them being genetically characterised, in turn, further helping to avoid ambiguities in species delimitation. *Lecithochirium microstomum* is an euryxenous parasite widely spread across the tropical and subtropical Atlantic and Pacific oceans [87]. This species is the most reported trematode species in marine fishes form Brazil. So far, fishes from 11 families of 8 orders are known to be the hosts of *L. microstomum* in the country [9,67]. Our report of *L. microstomum* in a triglid fish, *Pr. punctatus*, increased the host spectrum for this species. This species was previously reported five times in the same host, *T. lepturus*, and in the same locality in Brazil as in the present study [9]. Although numerous records of *L. microstomum* are available, there were no associated DNA sequence data for this species in Brazil. However, one 28S rDNA sequence of *L. microstomum* (KC985235) from *T. lepturus* collected in Gulf of Mexico, Mississippi, USA, by Calhoun et al. [88] is available and our isolate clustered in the same clade with it; the intraspecific sequence divergence was considered high (1.5%; 16 nt) when compared with the intraspecific sequence divergence observed in other *Lecithochirium* spp. (Figure 4 and Table S1). A morphological description was not provided with the published sequence, and therefore we cannot compare the morphology of the isolates. Based on morphological features and comparisons of the metrical data, our specimens agree with the descriptions of *L. microstomum*. An unidentified species of *Lecithochirium* (GenBank number MK648288) collected from *T. lepturus* in Veracruz, Mexico [89]—even with the lack of a reference morphological voucher for this sequence—is likely conspecific with *L. microstomum* found in the present study. The difference between the 28S rDNA sequences of our isolates and the isolate from Mexico was low, 0.47% (5 nt).

Molecular data showed that *L. synodi* and *L. microstomum* are closely related, although they belong to different morphological groups, i.e., “Synodi-group” and “Microstomum-group”. Morphological similarities are also remarkable, i.e., the absence of internal elevations of the ventral sucker, a tripartite seminal vesicle, the presence of a presomatic pit, obliquely tandem testes, and testes separated from the ventral sucker. A morphological feature that separates *L. synodi* and *L. microstomum* between different groups according to the key of Bray [68] is the type of presomatic pit (glandular in the “Synodi-group” and non-glandular in the “Microstomum-group”). Our specimens of *L. microstomum* possess a non-glandular presomatic pit. However, León-Regàgnon and co-authors [39] examined the type material of *L. microstomum* and concluded that the presomatic pit is glandular in the material of Chandler [70]. Due to the genus being specious, at the present stage it is not possible to identify which features are more phylogenetically important. Detailed morphological descriptions including molecular data are only available for 5 out of more than 100 nominal species of the genus [7,68,90].

Specimens tentatively identified as *L. muraenae* in our study were reported for the first time in *G. vicinus* and in Brazil. Prior to our study, this species was only known from the Pacific Ocean [80,81]. The record of this species in the Atlantic Ocean demonstrates that its geographical distribution is possibly wider than previously reported. *Lecithochirium muraenae* is a stenoxenous parasite infecting muraenid fishes. DNA sequence data for *L. muraenae* from muraenids in the Pacific Ocean were not available for comparison.

Interestingly, isolates of *Lecithochirium* cf. *muraenae* clustered distantly from other *Lecithochirium* spp. recorded in this study. It formed a well-supported clade with an unidentified species of *Lecithochirium* (ON614672) collected from *Octopus bimaculatus* in Mexico [90] and *Pu. cyanovitellosus* (the subfamily Pulmoverminae). Although the specimens from octopuses were immature, there are a couple of morphological similarities that we could observe when comparing to specimens of *Lecithochirium* cf. *muraenae*, these are: the position of the genital pore at the level of intestinal bifurcation, a blind caeca terminating in the body, symmetrical testes positioned laterally posterior to the ventral sucker, and an ovary closely posterior to dextral or sinistral testis. Morphologically, *Lecithochirium* cf. *muraenae* and *Pu. cyanovitellosus* conspicuously differ in body shape (plump, piriform vs. elongate, slender), the size of the ecsoma (well-developed vs. reduced), the shape and size of the seminal vesicle (short and straight vs. long and convoluted), and the position of the testes in relation to the ventral sucker and to each other (contiguous with the ventral sucker and symmetrical vs. separated from the ventral sucker and tandem). Additionally, these species parasitise different groups of hosts (fish vs. sea-snake) and were found in different sites in the hosts (stomach vs. lung). The published sequence of *Pu. cyanovitellosus* was not supplemented with either morphological descriptions of the isolate or an electronic voucher, and thus no data is available for morphological comparison of the actual isolates.

Based on their morphology, species of *L. muraenae* and *L. microstomum* were placed in the same group, the “Microstomum group” [68]. However, the comparative sequence analysis suggests that these species are rather genetically distant, and thus further revaluation of morphotype groups with DNA sequence data, including data for the type-species, *L. rufoviride* (Rudolphi, 1819), will aid in the identification of a valid composition of each group.

Currently, and including our data, several species of *Lecithochirium* are reported from Brazil, namely: *L. monticellii* (Linton, 1898), *L. imovacus* (Looss, 1907), *L. microstomum* Chandler, 1935, *L. texanum* (Chandler, 1941), *L. zeloticum* (Travassos, Teixeira de Freitas and Buhrnheim, 1966), *L. manteri* Teixeira de Freitas and Gomes, 1971, *L. perfidum* Gomes, Fabio and Rolas, 1972, *L. floridense* (Manter, 1934) and *L. synodi* Manter 1931 [7]. The record of *L*. cf. *muraenae* in the present study increased the diversity of *Lecithochirium* to 10 species in marine fish off the Brazilian coast, confirming that this genus is the most diverse in marine fishes in the region. Species of *Lecithochirium* are parasites of fishes from 14 families in the region [7,9].

The genus *Myosaccium* accommodates only two species which parasitise marine fishes of the Clupeidae and Carangidae, with their distribution restricted to the Atlantic and Pacific oceans [39]. Due to strong morphological similarity, Overstreet [46] suggested *M. opisthonemae* to be a synonym of *M. ecaude*. Although specimens of *M. opisthonemae* are smaller than those identified as *M. ecaude*, the main feature used to distinguish between them—the presence or absence of spines and polar filament on eggs—was demonstrated to be erroneous. Molecular genetic characterisation of smaller specimens is needed to assess the taxonomic status of *M. opisthonemae. Myosaccium ecaude* is a stenoxenous parasite and has previously been reported only in clupeid fishes, whereas *M. opisthonemae* is an euryxenous parasite and has been reported in clupeid and carangid fishes [9,16].

The relative relation of *M. ecaude* to *He. levinseni* in the clade containing the members of the subfamily Hemiurinae was rather surprising (Figure 4) because the genus *Myosaccium* currently belongs to the subfamily Aphanurinae. We found no convincing morphological characters, potentially justifying the move of *Myosaccium* into the Hemiurinae. The main feature differentiating *Myosaccium* and *Aphanurus* (type genus of the Aphanurinae) is the shape of the vitellarium (two distinct masses vs. one distinct mass, respectively), whereas *Hemiurus* and *Parahemiurus* possess vitellarium composed of two distinct masses. The taxonomic value of this character for subfamily classification is still to be confirmed.

The genus *Parahemiurus* is represented by 22 species [91]. In Brazil, only two species have been reported, the type-species *P. merus* and *P. anchoviae* Pereira and Vaz, 1930 [9]. *Parahemiurus merus* is an euryxenous parasite tending to be most frequently reported in temperate pelagic fishes and is rarely reported in the vast area covered by the tropical Indo-West Pacific region [65]. This species is the second most reported trematode species in marine fishes in Brazil. So far, fishes from 12 families of 9 orders are known to be the hosts of *P. merus* in Brazil [9]. Despite numerous records of *P. merus*, there were no DNA sequence data available for this species. Accordingly, and for the first time, we provided DNA sequences for the genus *Parahemiurus* and confirmed its position within the Hemiurinae based on molecular data.

According to the present and previous phylogenetic analyses of Atopkin et al. [11], Faltýnková et al. [92], and Sokolov et al. [12], it is evident that the subfamily classifications of the Hemiuridae, based entirely on morphological characters, needs to be reconsidered taking into account a wide range information sources, including morphological, molecular, and ecological data. Although the molecular library of the Hemiuridae is slowly growing, the lack of DNA sequences of numerous taxa, the limitation of data from single conservative molecular markers, and the absence of morphological vouchers of the actual isolates used to generate sequences, represents an impediment for the clarification of the taxonomic content of the family. Accordingly, such information is also imperative for understanding the value of the morphological features used to define subfamilies.

## 5. Conclusions

Based on morphological and molecular genetic analyses, we identified eight hemiurid species belonging to four genera parasitising marine fishes in Brazil. This is the first study to report phylogenetic relationships based on 28S rDNA sequences of members of three hemiurid genera, *Ectenurus*, *Myosaccium*, and *Parahemiurus*. These data contribute to the knowledge of the systematics and evolutionary history of the Hemiuridae which remains incomplete. In phylogenetic analysis, the aphanurines *Myosaccium* and *Aphanurus* were rendered paraphyletic. This data casts doubts over the traditional subfamily classification based on the morphology of the members from these genera, which will need to be rearranged in the future when a higher number of sequenced hemiurid taxa will be available. The record of the specimens tentatively identified as *Lecithochirium muraenae* increases the number of hemiurid species off the Brazilian coast. The first records of digenean trematodes in the fishes *Anisotremus virginicus* and *Decapterus punctatus* in the country, indicates the relatively poor coverage of Brazilian marine digenean fauna. Studies on marine fish trematodes using morphological and molecular datasets remain in their infancy in Brazil. Our findings highlight the importance of exploring this region, which is considered as a parasite biodiversity hotspot [93], and the need to describe digenean diversity using integrative data.

## Figures and Tables

**Table 3:** Comparative metrical data of *Parahemiurus merus* (Linton, 1910).

Source	Present Study		León-Règagnon et al. [39]		Linton [49]	Vaz and Pereira [63]	Timi et al. [64]		Bray [65]
Locality	Atlantic Ocean, Rio de Janeiro, Brazil		Pacific Ocean, Jalisco, Mexico		Atlantic Ocean, Florida, USA	Atlantic Ocean, São Paulo, Brazil	Atlantic Ocean, Argentinean and Uruguayan coasts		Atlantic, Indian, and Pacific oceans
Host	*Harengula clupeola*, *Sardinella brasiliensis*		Balsitidae, Clupeidae, Engraulidae, Haemulidae		*Sardinella aurita*	*Sardinella aurita*	*Eugraulis anchoita*		Carangidae, Clupeidae, Haemulidae, Merlucciidae, Pomatomidae, Salmonidae, Scorpaenidae, Sparidae
	Range (*n* = 4)	Mean	Range (*n* = 4)	Mean	Range (*n* = 1)	Range (*n* = NP)	Range (*n* = 20)	Mean	Range (*n* = NP)
Body length	704–1619	1067	890–1580	1100	2940	1510–1930	720–1441	987	800–2990
Body width	146–407	260	210–480	290	500	360–400	120–247	171	210–570
Ecsoma length	257−612	435	180–680	420	–	480–500	–	–	Extend to 1020
Total length	1061–2231	1646	–	–	–	–	–	–	–
Forebody length	118−360	207	–	–	–	–	–	–	–
Hindbody length	479−2062	1083	–	–	–	–	–	–	–
Pre-oral lobe length	7−22	12	–	–	–	–	8–36	15	8–50
Oral sucker length	53−83	65	40–60	50	90	80	50–80	63	36–83
Oral sucker width	53−86	68	60–70	60	–	80	53–80	66	51–82
Pharynx length	39−57	47	–	–	70	40	33–50	41	32–64
Pharynx width	37−58	44	–	–	–	40	30–50	40	32–51
Oesophagus length	10–37	26	–	–	–	–	–	–	–
Ventral sucker length	101−182	133	120–150	140	210	180–200	106–165	133	101–170
Ventral sucker width	103−186	139	100–190	130	–	–	99–200	137	124–170
DIBAE	122−187	147	–	–	–	–	–	–	–
DTVS	114–377	203	–	–	–	–	–	–	–
Anterior testis length	45−82	70	–	–	–	60–120	40–83	64	40–152
Anterior testis width	34−89	61	–	–	–	–	50–132	78	68–145
Posterior testis length	39–87	69	–	–	–	–	36–90	74	48–170
Posterior testis width	42–74	61	–	–	–	–	46–125	88	64–170
Post-testicular region length	300–886	516	–	–	–	–	–	–	–
Seminal vesicle length	58−177	107	–	–	–	–	50–96	73	48–278
Seminal vesicle width	27−105	58	–	–	–	–	36–63	46	41–130
Sinus-sac length	128–256	176	–	–	–	120–180	–	–	95–220
Sinus-sac width	14–30	19	–	–	–	–	–	–	–
Ovary length	32−93	65	–	–	–	80–160	40–102	73	47–234
Ovary width	40−129	87	–	–	–	–	76–149	104	80–233
Vitellarium length (dextral)	42–131	75	–	–	–	80–180	56–132	97	–
Vitellarium width (dextral)	43–93	66	–	–	–	–	43–116	80	–
Vitellarium length (sinistral)	38–129	72	–	–	–	–	59–178	118	–
Vitellarium width (sinistral)	46–119	77	–	–	–	–	50–142	99	–
DBOT	114–377	203	–	–	–	–	–	–	–
Egg length	22–27	25	20–25	22	27	24	24–32	28	20–32
Egg width	7–11	9	9–13	11	10	10–14	8–12	10	–
Body length/body width	1:2.83–5.65	1:4.32	–	–	–	–	–	–	–
Oral/ventral sucker width	1:1.94−2.16	1:2.03	1:2.90	–	–	–	1:1.71–2.5	1:1.58	1:1.76–2.58
Ecsoma/body length, %	32−38	35	–	–	–	–	–	–	–
Forebody/body length, %	14−32	19	–	–	–	–	–	–	6–19
Post-testicular region/body length, %	37–55	46	–	–	–	–	–	–	–

DIBAE = distance from intestinal bifurcation to anterior extremity, DTVS = distance from testes to ventral sucker, DBOT = distance between ovary and testes, NP = not provided.

**Table 4:** Comparative metrical data of *Lecithochirium microstomum* Chandler, 1935.

Source	Present Study		León-Règagnon et al. [39]		Timi et al. [64]		Wallet and Kohn [66]	Chandler [70]	Teixeira de Freitas and Kohn [71]
Locality	Atlantic Ocean, Rio de Janeiro, Brazil		Atlantic Ocean, Chamela Bay, Mexico		Atlantic Ocean, Argentinean and Uruguayan coasts		Atlantic Ocean, Rio de Janeiro, Brazil	Atlantic Ocean, Texas, USA	Atlantic Ocean, Rio de Janeiro, Brazil
Host	*Prionotus punctatus*, *Trichiurus lepturus*		Carangidae, Engraulidae, Fistularidae, Lutjanidae, Scombridae		*Engraulis anchoita*		*Trichiurus lepturus*	*Trichiurus lepturus*	*Trichiurus lepturus*
	Range (*n* = 6)	Mean	Range (*n* = 10)	Mean	Range (n = 20)	Mean	Range (*n* = 3)	Range (*n* = NP)	Range (*n* = NP)
Body length	3104–4292	3734	1830−3920	2940	744−1592	1034	3640–4270	2750–4800	3330−5170
Body width	641–769	690	450–810	600	224−496	323	570−840	875–1000	750−1170
Ecsoma length	974–1351 (*n* = 4)	1135	−	−	112−384	219	−	1000	−
Total length	4078–6418 (*n* = 4)	5084	−	−	−	−	−	3760 (*n* = 1)	−
Forebody length	520−732	635	−	−	−	−	−	−	−
Hindbody length	2215−3119	2616	−	−	−	−	−	−	−
Pre-oral lobe length	33−73	51	−	−	11−40	21	−	−	−
Oral sucker length	170−198	184	90−180	130	88−145	120	140−150	140–200	170−220
Oral sucker width	164−202	180	110−180	150	97−160	121	180−190	−	200−250
Pharynx length	95−106	99	−	−	53−78	62	190−210	70–110	70−120
Pharynx width	93−111	99	−	−	46−82	65	−	−	100–120
Oesophagus length	38–84	56	−	−	−	−	−	−	−
Ventral sucker length	413−555	485	280−540	450	155−309	208	470−480	365−540	330−610
Ventral sucker width	450−536	502	310−490	440	153−296	208	510−610	−	400−640
DIBAE	296−350	334	−	−	−	−	−	−	−
DTVS	209−409	304	−	−	−	−	−	−	−
Anterior testis length	244−302	274	−	−	23−118	55	180−230	−	200−370
Anterior testis width	157−243	194	−	−	25−141	62	200−280	−	180−420
Posterior testis length	270–305	288	−	−	−	−	−	−	230−380
Posterior testis width	187–242	212	−	−	−	−	−	−	250−450
Post-testicular region	1295–2335	1711	−	−	−	−	−	−	−
Seminal vesicle length	188−462	360	−	−	65−153	91	−	−	670−900
Seminal vesicle width	65−236	164	−	−	25−74	43	−	−	170−230
Sinus-sac length	139–221	172	−	−	−	−	−	−	−
Sinus-sac width	103–154	120	−	−	−	−	−	−	−
Ovary length	184−271	232	−	−	32−99	53	150−180	−	220−400
Ovary width	239−292	260	−	−	40−147	71	200−280	−	330−430
Vitellarium length	246–357	314	−	−	−	−	−	−	−
Vitellarium width	211–442	285	−	−	−	−	−	−	−
DBOT	467–600	532					−	−	−
Egg length	18–26	21	15–21	18	15–19	16	19−23	16	15−22
Egg width	10–15	11	9−13	11	8–11	9	9−14	12	11−13
Body length/body width	1:4.84–5.93	1:5.41	−	−	−	−	−	−	−
Oral/ventral sucker width	1:2.53−3.12	1:2.82	1:2.9−4.6	1:3.5	−	−	1:3.0−3.30	1:2.50–2.80	1:2.12−2.97
Ecsoma/body length, %	29−31 (*n* = 4)	30	−	−	−	−	−	−	−
Forebody/body length, %	16−20	17	−	−	−	−	−	−	−
Post-testicular region/body length, %	39–54	45	−	−	−	−	−	−	−

DIBAE = distance from intestinal bifurcation to anterior extremity, DTVS = distance from testes to ventral sucker, DBOT = distance between ovary and testes, NP = not provided.

**Table 5:** Comparative metrical data of *Lecithochirium* spp.

Species	*Lecithochirium* cf. *muraenae*		*Lecithochirium muraenae* Manter, 1940	*Lecithochirium* sp.	*Lecithochirium monticellii* Linton (1898)		*Lecithochirium monticellii*	
Source	Present study		Manter [80]	Present study	França et al. [15]		França et al. [15]	
Locality	Atlantic Ocean, Rio de Janeiro, Brazil		Pacific Ocean, Cape Elena, Ecuador	Atlantic Ocean, Rio de Janeiro, Brazil	Atlantic Ocean, Rio de Janeiro, Brazil		Atlantic Ocean, Rio de Janeiro, Brazil	
Host	*Gymnothorax vicinus*		*Muraena clepsydra*	*Trichiurus lepturus*	*Trichiurus lepturus*		*Trichiurus lepturus*	
	Range (*n* = 3)	Mean	(*n* = 3)	(*n* = 1)	Range (*n* = 10)		Range (*n* = 10)	Mean
Body length	2206–2415	2276	3684–5359	2607	2020–4020	2840	1020–1900	1400
Body width	610–889	742	958–1120	694	270–520	400	220–500	350
Ecsoma length	520 (*n* = 1)	520	–	–	150–1520	580	170–500	250
Total length	2726 (*n* = 1)	2726	–	–	–	–	–	–
Forebody length	455−530	499	756–1188	552	–	–	–	–
Hindbody length	1353−1466	1394		1608	–	–	–	–
Pre-oral lobe length	12−33	22		60	–	–	–	–
Oral sucker length	135−163	149	300–375	173	60–140	100	70–130	100
Oral sucker width	154−197	169	–	182	–	–	–	–
Pharynx length	85−95	90	120–170	111	–	–	–	–
Pharynx width	83−112	94	75–120	92	–	–	–	–
Oesophagus length	19–42	30	–	108	–	–	–	–
Ventral sucker length	366−454	398	715–883	424	210–410	230	150–310	260
Ventral sucker width	358−465	403	–	447	–	–	–	–
DIBAE	272−317	287	–	393	–	–	–	–
DTVS	−	−	−	204	−	−	−	−
Anterior testis length	116−120	114	–	91	–	–	–	–
Anterior testis width	98−113	108	–	72	50–200	120	50–130	80
Posterior testis length	96–103	101	–	117	50–190	90	50–110	90
Posterior testis width	111–128	119	–	100	60–210	140	60–150	80
Post-testicular region	1251–1383	1304	–	1273	60–170	110	60–130	80
Seminal vesicle length	267−321	287	–	217	110–340	200	50–170	100
Seminal vesicle width	109−131	118	–	87	–	–	–	–
Sinus-sac length	116–129	123	–	179	50–100	70	20–100	–
Sinus-sac width	45–46	46	–	46	–	–	–	60
Ovary length	75−89	81	–	100	50–220	110	50–130	70
Ovary width	99−162	127	–	158	60–200	130	60–150	100
Vitellarium length	78–130	101	–	344	–	–	–	–
Vitellarium width	98–161	124	–	230	–	–	–	–
DBOT	48–72	63	–	291	–	–	–	–
Egg length	10–14	12	15–19	11–15	23–28	24	15–28	18
Egg width	9–13	11	10–12	9–11	10–13	11	10–13	10
Body length/body width	1:2.72–3.62	1:3.12	–	1:3.76	–	–	–	–
Oral/ventral sucker width	1:2.32−2.47	1:2.38	1:3.70	1:2.46	1:1.95–2.41	–	1:1.73–2.10	–
Ecsoma/body length, %	24 (*n* = 1)	24	–	–	–	–	–	–
Forebody/body length, %	21−24	22	–	21	–	–	–	–
Post-testicular region/body length, %	57–58	57	–	49	–	–	–	–

* DIBAE = distance from intestinal bifurcation to anterior extremity, DTVS = distance from testes to ventral sucker, DBOT = distance between ovary and testes.

## Data Availability

The data generated in this study are available from the corresponding author upon request.

## Supplementary Materials

The following supporting information can be downloaded at: https://www.mdpi.com/article/10.3390/ani12233355/s1, Figure S1: Photomicrographs of the molecular vouchers of fish; Table S1: Pairwise comparisons of genetic distances of the Hemiuridae based on 28S rDNA sequences (957 nt long alignment); Table S2: Nucleotide comparison of the partial ITS2 sequences of *Lecithochirium* spp. based on 416 nt long alignment. Table S3: Nucleotide comparison of *cox*1 sequences of *Lecithochirium* spp. based on 443 nt long alignment.
