# Supplementary material for: Hemiurid Trematodes (Digenea: Hemiuridae) from Marine Fishes off the Coast of Rio de Janeiro, Brazil, with Novel Molecular Data
Source: Animals (Basel). 2022 Nov 29;12(23):3355. doi: 10.3390/ani12233355 (PMC9741374; doi:10.3390/ani12233355)
Supplement: Supplementary file 1 [file animals-12-03355-s001.zip › Pantoja & Kudlai Figure S1 Fish Vouchers .pdf]

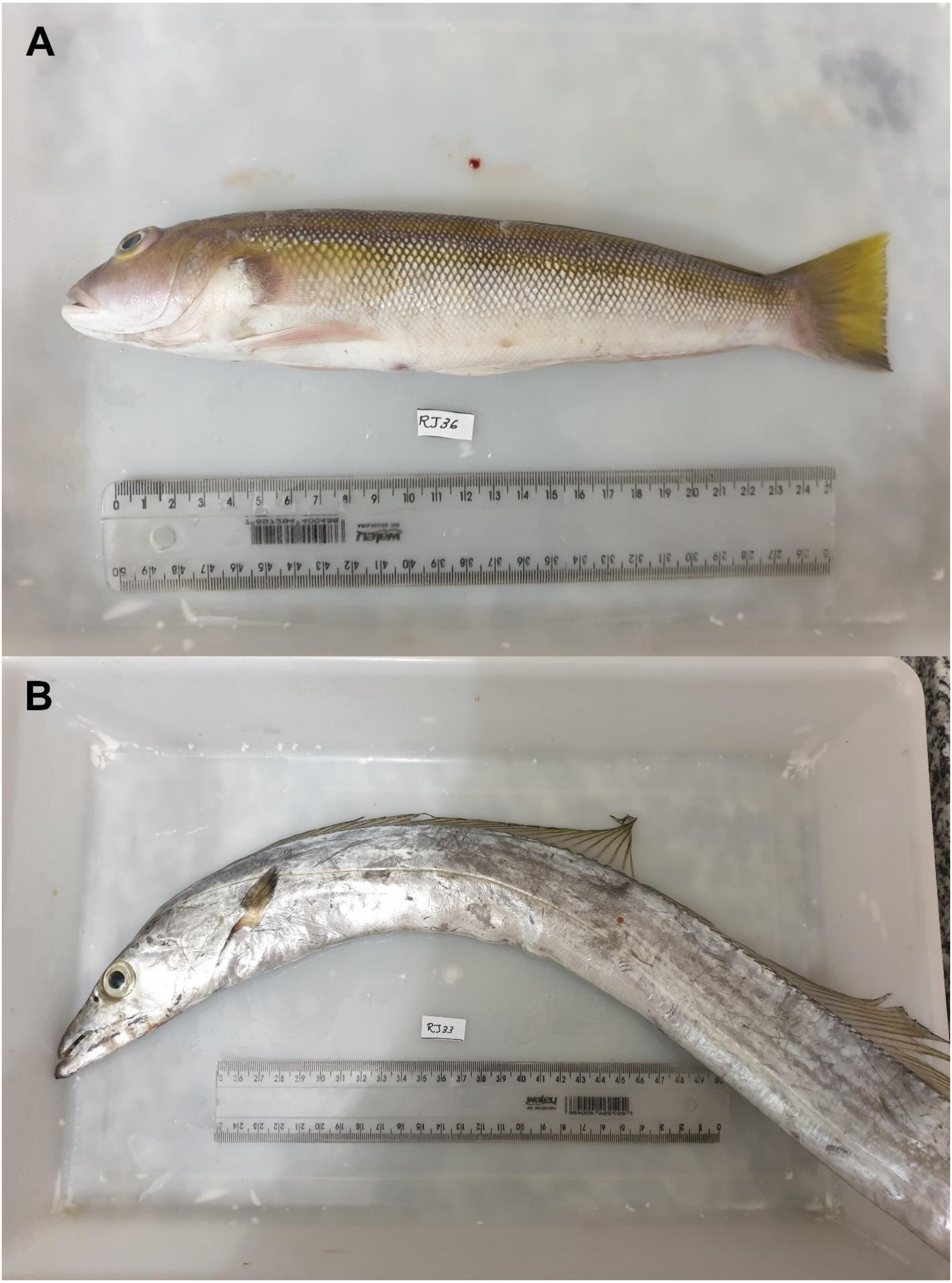

Figure S1. Photomicrographs of the molecular vouchers of fish: a, *Pseudopsercis numida* Miranda Ribeiro (OP925860); b, *Trichiurus lepturus* (Linnaeus) (OP905634)
