# Supplementary material for: Hemiurid Trematodes (Digenea: Hemiuridae) from Marine Fishes off the Coast of Rio de Janeiro, Brazil, with Novel Molecular Data
Source: Animals (Basel). 2022 Nov 29;12(23):3355. doi: 10.3390/ani12233355 (PMC9741374; doi:10.3390/ani12233355)
Supplement: Supplementary file 1 [file animals-12-03355-s001.zip › Pantoja & Kudlai Table S2 Pairwise sequence comparison ITS2.pdf]

Table S2. Nucleotide comparison of the partial ITS2 sequences of *Lecithochirium* spp. based on 416 nt long alignment. P-distance (%) is given below diagonal and the number of variable nucleotides above diagonal.

		1	2	3	4	5	6
1	OP918139 *Lecithochirium synodi*		0	8	56	58	100
2	OP458338 *Lecithochirium synodi*	0		8	56	58	100
3	OP918137 *Lecithochirium microstomum*	1.92	1.92		60	62	98
4	KU527429 *Lecithochirium floridense*	13.53	13.53	14.49		11	90
5	OP458339 *Lecithochirium floridense*	14.01	14.01	14.95	2.65		89
6	OP918139 *Lecithochirium* cf. *muraenae*	24.33	24.33	23.84	21.95	21.71	
