# Supplementary material for: Hemiurid Trematodes (Digenea: Hemiuridae) from Marine Fishes off the Coast of Rio de Janeiro, Brazil, with Novel Molecular Data
Source: Animals (Basel). 2022 Nov 29;12(23):3355. doi: 10.3390/ani12233355 (PMC9741374; doi:10.3390/ani12233355)
Supplement: Supplementary file 1 [file animals-12-03355-s001.zip › Pantoja & Kudlai Table S3 Pairwise sequence comparison cox1.pdf]

Table S3. Nucleotide comparison of *cox*1 sequences of *Lecithochirium* spp. based on 443 nt long alignment. P-distance (%) is given below diagonal and the number of variable nucleotides above diagonal.

		1	2	3	4	5	6	7	8
1	OP418194 *Lecithochirium synodi*		1	2	66	70	88	88	111
2	OP918024 *Lecithochirium synodi*	0.23		1	67	71	89	89	111
3	OP918026 *Lecithochirium synodi*	0.45	0.23		68	70	90	90	112
4	OP918021 *Lecithochirium microstomum*	14.90	15.12	15.35		5	102	102	111
5	OP918022 *Lecithochirium microstomum*	15.80	16.03	15.80	1.13		104	104	113
6	OP418195 *Lecithochirium floridense*	19.86	20.09	20.32	23.02	23.48		0	115
7	OP918025 *Lecithochirium floridense*	19.86	20.09	20.32	23.02	23.48	0		115
8	OP918023 *Lecithochirium muraenae*	25.06	25.06	25.28	25.06	25.51	25.96	25.96	
